# Photoinduced Porcine Gelatin Cross-Linking by Homobi- and Homotrifunctional Tetrazoles

**DOI:** 10.3390/gels7030124

**Published:** 2021-08-20

**Authors:** Luca Vaghi, Mauro Monti, Marcello Marelli, Elisa Motto, Antonio Papagni, Laura Cipolla

**Affiliations:** 1 Dipartimento di Scienza dei Materiali, Università degli Studi di Milano—Bicocca, via R. Cozzi 55, 20125 Milano, Italy; mauro.monti@unimib.it; 2 Istituto di Scienze e Tecnologie Chimiche “Giulio Natta”, CNR-SCITEC, Sede Fantoli, via Fantoli 16/15, 20138 Milano, Italy; marcello.marelli@scitec.cnr.it; 3 Dipartimento di Biotecnologie e Bioscienze, Università degli Studi di Milano—Bicocca, Piazza della Scienza 2, 20126 Milano, Italy; e.motto2@campus.unimib.it

**Keywords:** gelatin, hydrogel, photochemistry, tetrazoles, nitrilimine, chemical cross-linking, natural polymers

## Abstract

Gelatin is a costless polypeptide material of natural origin, able to form hydrogels that are potentially useful in biomaterial scaffold design for drug delivery, cell cultures, and tissue engineering. However, gelatin hydrogels are unstable at physiological conditions, losing their features only after a few minutes at 37 °C. Accordingly, treatments to address this issue are of great interest. In the present work, we propose for the first time the use of bi- and trifunctional tetrazoles, most of them unknown to date, for photoinduced gelatin cross-linking towards the production of physiologically stable hydrogels. Indeed, after UV-B irradiation, aryl tetrazoles generate a nitrilimine intermediate that is reactive towards different functionalities, some of them constitutively present in the amino acid side chains of gelatin. The efficacy of the treatment strictly depends on the structure of the cross-linking agent used, and substantial improved stability was observed by switching from bifunctional to trifunctional cross-linkers.

## 1. Introduction

Proteins are valuable polymers, serving as biomaterials in applications that revolutionized regenerative medicine, tissue regeneration [1,2], and drug delivery [3] in recent decades. Proteins derived from extracellular matrices (ECM, i.e., collagen, fibronectin, laminin [4,5]) offer several advantages over non-natural materials, since they support cell growth and tissue regeneration, reduce undesired side effects, and are biodegradable and biocompatible. However, the main limiting factor to their use is their availability, which impacts costs, and effective substitutes for ECM proteins are thus highly desirable. In this framework, gelatin is a widespread natural polymer used for the design of innovative biomaterials as an ECM protein substitute [6] and as a drug delivery vehicle [7], already used in well-established applications in the pharmaceutical, cosmetic, and food industries [8]. Gelatin is obtained from collagen by chemical or enzymatic hydrolysis, or heat denaturation, and subsequently manufactured with different advanced techniques such as electrospinning, 3D-printing, solvent casting, rapid prototyping, and bioprinting in different scaffolds with the desired morphology, topography, mechanical, and biological properties [9,10]. Gelatin offers the advantage of being readily and widely available, water soluble compared to other ECM proteins, and suitable for cell attachment in regenerative medicine applications. One disadvantage of gelatin in the fabrication of biomaterials is its poor mechanical strength [11,12,13,14,15], which may be improved by cross-linking. Cross-linking can be achieved either by chemical, enzymatic, or physical methods [16,17,18,19]. Chemical cross-linking offers advantages over physical methods, affording stable linkages and reproducibility. To achieve chemical cross-linking, either amino acid side chains [20,21] or additional functional groups [22] suitably grafted to the protein can be employed. Regioselective and bioorthogonal chemistries can be used in order to fine-tune cross-linking and, by extension, the final properties of the biomaterial [23]. Bioorthogonal reactions rely on introduction into the polymers of functional groups different from those found in amino acid side chains, able to chemoselectively react in mild conditions with high yields. Among them, the so-called click chemistry [24,25,26,27], based, for example, on Huisgen-type cycloaddition [28], Staudinger reaction [29,30], Diels–Alder [31,32,33], thiol–ene addition [34,35], and carbonyl/oxime-hydrazone chemistry [36,37], has been proposed over recent years. The main drawback of bioorthogonal reactions arises from the need for a two-step process, involving first the introduction of orthogonal functional groups, either by chemical or enzymatic reactions [38] or protein engineering [39] approaches. On the other hand, reactions based on the direct cross-linking of amino acid side chains may suffer from the release of (toxic) by-products, which are difficult to be eliminate. Several examples of gelatin cross-linking, both by click chemistry [40,41,42,43,44] and direct cross-linking based on intrinsic amino acid reactivity, were proposed. Direct cross-linking can be obtained by homo- or ethero-difunctional cross-linking agents of natural origin, such as citric acid [45], and genipin [46], or non-natural ones, including bisvinyl sulfonemethyl (BVSM) [47], 1,4-butanediol diglycidyl ether (BDDGE) [16], triazolinediones [48], and the widely-used glutaraldehyde [49,50].

Despite great advances in recent years, the search for additional chemistries that can be applied to amino acid side chain reactivity in order to achieve chemoselectivity, a one-step reaction, and no by-products, is still on-going.

**Scheme 1:**
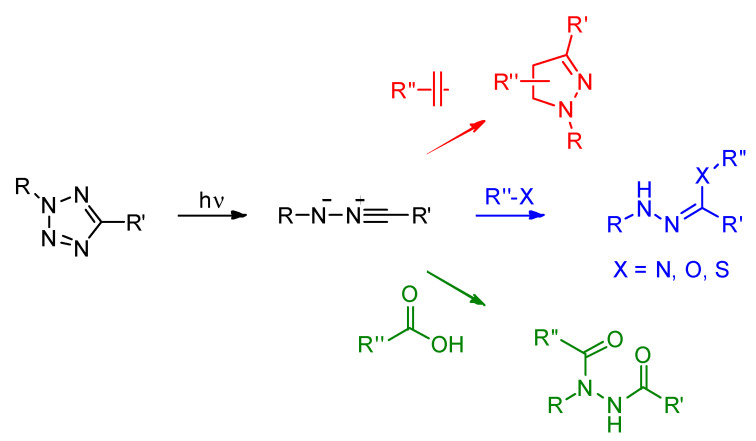
Photoinduced nitrilimine generation and reactivity towards functional groups.

Tetrazoles were recently used as photoactivable precursors for the reaction towards biological nucleophiles [51,52,53]. Upon UV irradiation, the tetrazole moiety release a nitrogen molecule to produce a highly reactive nitrilimine intermediate, able to react with a plethora of functional groups (Scheme 1) [54,55,56].

Even if it is possible to direct the reactivity of nitrilimines to the desired functionality by varying the reaction conditions [52], complete selectivity is almost unattainable, and unpredictable results were observed with complex biological substrates [53]. This behavior could be detrimental to achieving a site-selective functionalization or bioconjugation. However, it may be ideal as an effective cross-linking strategy to achieve improvements in the macroscopic characteristics of biological materials, such as gelatin. Intrigued by this possibility and exploiting our expertise in the synthesis of heterocyclic compounds [57,58,59,60], we prepared a set of bi- and trifunctional tetrazoles and applied them for the first time in the photoinduced cross-linking of porcine skin gelatin.

## 2. Results and Discussion

### 2.1. Synthesis of Cross-Linking Agents

**Scheme 2:**
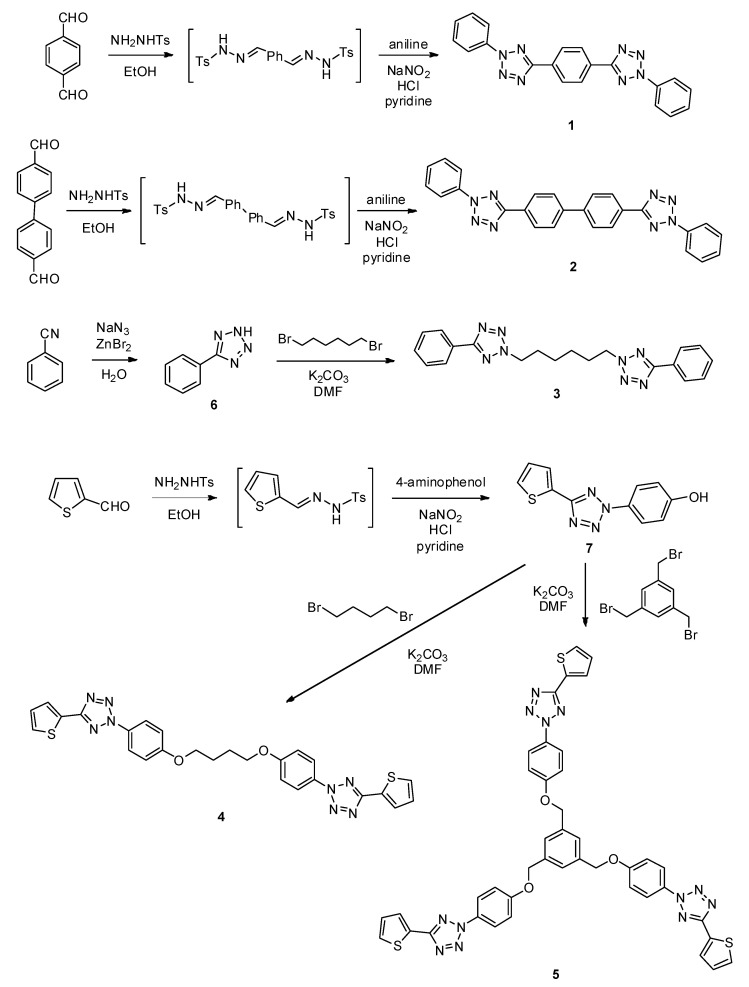
Synthesis of the cross-linking agents **1**–**5** used in this study.

Four homo-bifunctional and one homo-trifunctional tetrazoles were synthesized, four of them unknown to date, to be used in the photoinduced cross-linking of gelatin (Scheme 2).

Cross-linking agent **1** was previously reported [61,62], however for its preparation in this study, a different synthetic strategy was used, involving the treatment of terephthalaldehyde bistosylhydrazone with diazonium aniline salt [63]. By the same methodology, using 4,4′-diformylbiphenyl instead of terephthalaldehyde tetrazole, **2** was easily obtained. The preparation of **3**, **4,** and **5** involved the synthesis of the tetrazole moiety, then, via alkylation with the suitable di- and tri-bromo derivatives, the desired compound was obtained; i.e., 5-phenyl-2H-tetrazole **6** [64] was treated with 1,6-dibromohexane in the presence of K_2_CO_3_ in the case of **3**, while 4-(5-(thiophen-2-yl)-2H-tetrazol-2-yl)phenol **7**, achieved by treatment of 2-thiophenecarboxyaldehyde tosylhydrazone with the diazonium salt of 4-aminophenol, was reacted with 1,4-dibromobutane and 1,3,5-tris(bromomethyl)benzene in presence of K_2_CO_3_ to afford **4** and **5**, respectively. Detailed synthetic procedures are reported in the Supplementary Materials.

### 2.2. PhotoInduced Gelatin Cross-Linking

The amino acid composition of porcine gelatin [65] shows the significant presence of glutamic acid (7.2 mol%) and aspartic acid (4.7 mol%) residues, whose carboxylic acid side chains could be targeted by photoactivated tetrazoles [51,52], as well as traces of hystidine (0.6 mol%), terminals COOH and NH_2_, and other possible reactive peptide side chain sites among the polypeptide network of gelatin. The perfect solubilization of both gelatin and selected cross-linking agent in the reaction media is desirable, in order to allow the cross-linker to reach the reactive sites and achieve reproducible cross-linking results. Dimethyl sulfoxide (DMSO) was selected as the media for photoinduced cross-linking procedures, as it is able to solubilize both gelatin (up to 12 mg/mL) and cross-linkers **1**–**5**. Thus, a set of experiments at increasing linker:gelatin ratios was planned, i.e., 100 mg of dry gelatin in the presence of 1, 2, 5, and 10 µmol of cross linker (the use of 20 µmol results in a heterogeneous mixture for almost every compound). Before the UV irradiation of gelatin-tetrazole homogeneous DMSO solution, it is important to remove oxygen from the reaction environment, together with a reduction in the UV-C component, in order to avoid UV-related oxidative damage to the polypeptide network [66,67,68]. Therefore, the solutions were bubbled with flowing nitrogen for 15 min before irradiation and maintained under an inert atmosphere during the photoinduced reaction. Standard borosilicate glassware was used, as this material transmit over 90% of UV light with wavelengths greater than 300 nm, while partially filtering the undesirable UV-C [69], and at the same time being reliable in the photoactivation of tetrazoles [55]. No absorption interference was expected from gelatin (as it is totally transparent to wavelengths > 240 nm [70]) or DMSO (as it absorbs no UV-B wavelengths, with a cutoff around 260 nm). The photoactivation of tetrazole is reported to be highly effective. Kinetic studies of the photoinduced nucleophilic attack demonstrated that addition between tetrazoles and COOH groups could be completed in less than 10 min, unless a strong electron-withdrawing group is present on either the C or N moiety of 2,5-tetrazoles [51,56], which is not the case for compounds **1**–**5**. The solutions, under vigorous stirring, were then irradiated with two coupled low-pressure mercury UV-B lamps with a peak at 310 nm, for 10 min.

The workup of the reaction mixture is fundamental to remove any trace of unreacted cross-linker, byproducts, and organic solvents from the hydrogels. Cross-linked gelatin precipitation from reaction media was the method of choice. The solvent used for the precipitation of the gelatin from the reaction medium (DMSO) must afford a satisfactory quantity of recovered gel without changing its properties, be miscible with DMSO for the precipitation and with H_2_O to be subsequently removed, and able to solubilize unreacted cross-linker and possible byproducts. Thus, methanol, ethanol, 2-propanol, and tetrahydrofuran (THF) were tested. All are effective for the precipitation of the hydrogels (2-propanol afforded lower yield); however, alcohols were unable to dissolve tetrazoles **1**–**5**, and consequently THF was selected for the workup step. Therefore, the treated gelatins were precipitated from the reaction solutions by 1:1 *v*/*v* addition of THF, and the obtained hydrogels were subsequently washed first with THF to remove unreacted crosslinkers and byproducts, then washed multiple times with H_2_O, and finally kept in H_2_O overnight to remove any traces of DMSO and THF by diffusion. The recovered hydrogels were finally freeze-dried for the subsequent experiments.

### 2.3. Thermal Stability of Prepared Hydrogels

**Figure 1:**
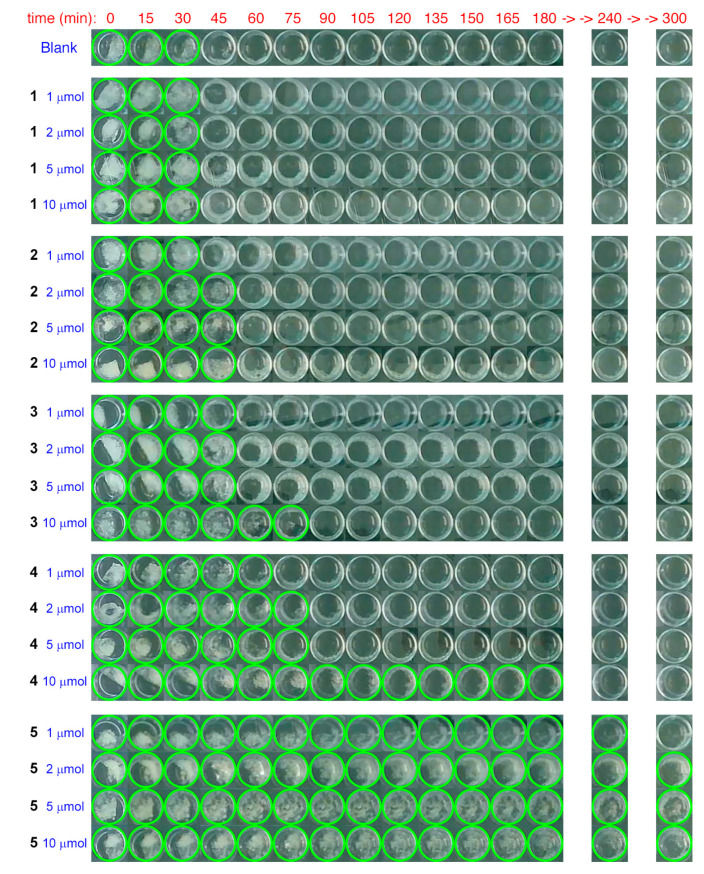
Time evolution of prepared hydrogels at 37 °C. Images of the sealed wells were caught every 15 min. When hydrogel consistence is maintained the well is circled in green (original video frames are presented in the Supplementary Materials).

One of the major disadvantages of gelatin-based hydrogels is the poor resistance to physiological conditions (i.e., at 37 °C they almost immediately dissolve). Thus, the prepared dry gelatin specimens were rehydrated with PBS buffer (pH = 7.4) and sealed and placed in a 37 °C thermostated chamber to test their thermal stability in comparison to blank gelatin treated in the same way, but without the presence of any cross-linker. The time evolution of hydrogels is depicted in Figure 1.

Blank gelatin resists 30 min in these conditions before complete dissolution. No stability improvements were observed for all samples treated with **1** (1, 2, 5, and 10 µmol) and **2** at the lowest amount (1 µmol), while a slight 15 min increase in thermal resistance was obtained using **2** at higher amounts (2, 5, and 10 µmol) and **3** (1, 2, and 5 µmol). The first significant differences were noted with gelatin treated with **3** at 10 µmol, which remained stable for 75 min, and with cross-linker **4** that afforded 60, 75, and 180 min of thermal resistance at increasing amounts of 1, 2/5, and 10 µmol, respectively. Switching to trifunctional cross-linker **5,** a substantial increment in thermal stability was observed, even at the lowest amount tested: at a 1 µmol/100 mg gelatin ratio, the specimen maintained hydrogel consistence up to 240 min, while at higher cross-linker amounts (2, 5, and 10 µmol), no dissolution was observed over 24 h.

These results may be explained by three main issues considering cross-linking agents **1**–**5**, namely their structure, photoactivation, and reactivity.

Concerning the structure, there are two points to consider: first, the distance between the two functional groups reacting with amino acid side chains should be high enough for cross-linking (usually referred to as cross-linker length). Among the cross-linkers **1**–**5**, compound **1** has the shortest length, thus possibly explaining its poor performance in the cross-linking reaction. Second, conformational flexibility may improve the ability of linkers to catch reactive functional groups in the complex, tridimensional structure of gelatin: in this respect, **1** and **2** have limited conformational freedom compared to **3**, **4**, and **5**.

As anticipated, photoactivation of 2,5-tetrazoles is reported to be highly effective [51,56], and 10 min of UV irradiation with a power (30 W) five time greater than that used in the literature (6 W) was assumed as a sufficient time for gelatin cross-linking to occur. In order to better characterize the reactivity of the system, λ_max_ of **1**–**5** was determined to check if they fit the settled irradiation window: **1**, **2**, **4**, and **5** displayed the maximum absorbance around 300 nm, with a perfect fit of **2** at 312 nm, while the absorption of **3,** where the aryl substituent on tetrazole nitrogen is replaced by an alkyl chain, was at more energetic wavelengths with a λ_max_ below 265 nm. In this case, a limited portion of the irradiating setup was useful for tetrazole activation.

A combination of the above-mentioned considerations resulted in a better performance of **4** and **5** in respect to **1**, **2**, and **3**. However, a crucial point to be considered is the competing reaction of nitrilimine intermediates towards gelatin COOH groups and the decomposition pathways. The reaction of photoactivated 2,5-diaryltetrazoles in the presence of a large excess of unhindered carboxylic acids affords *N’*-acyl-*N’*-phenylbenzohydrazides in up to 80% yields [52,55]. However, increased steric hindrance near the COOH functionality and/or lowered excess of the carboxylic acid cause a sensible drop in yield. Therefore, the application of this chemistry to gelatin, whose COOH functionalities may be hindered by the complex polypeptide structure and are not present in large excess in respect to the cross-linkers, may be much less effective than expected. Moreover, a cross-link requires that both the reactive moieties of a bifunctional reagent react with the target functionalities of the polymeric substrate. Accordingly, emerged the great advantage of trifunctional cross-linker **5** compared to bifunctional **1**–**4**: when the first nitrilimine links to gelatin, **5** has two reactive sites for cross-linking, significantly increasing the chances of success.

Due to their poor to null thermal stability increment, specimens obtained by treatment of gelatin with compounds **1**, **2**, and **3** were not investigated further, while further characterization of specimens treated with **4** and **5** was performed.

### 2.4. Characterization of Gelatin Treated with **4** and **5**

#### 2.4.1. Scanning Electron Microscopy Micrographs

**Figure 2:**
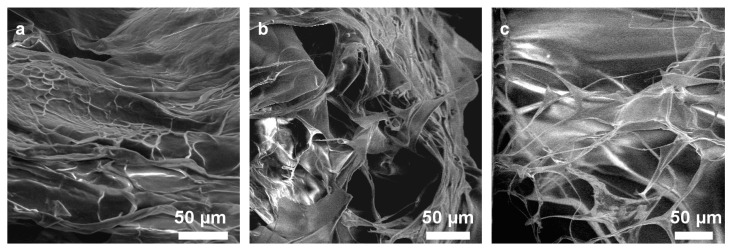
Representative SEM micrograph of dry gelatin samples: (**a**) pristine gelatin; (**b**) gelatin treated with **4** 10 μmol; (**c**) gelatin treated with **5** 10 μmol.

Low-vacuum scanning electron microscopy (SEM) was used to investigate the morphological structure of dry specimens after treatment with **4** and **5** at 10 µmol (Figure 2). Changes in the morphology of treated samples with respect to the reference blank were observed. In detail, the gelatin treated with cross-linkers **4** and **5** showed an increase in the apparent porosity, with a more open texture, the formation of a structured network, and a less solid appearance. These effects are more marked with cross-linker **5**.

#### 2.4.2. Swelling Properties

**Figure 3:**
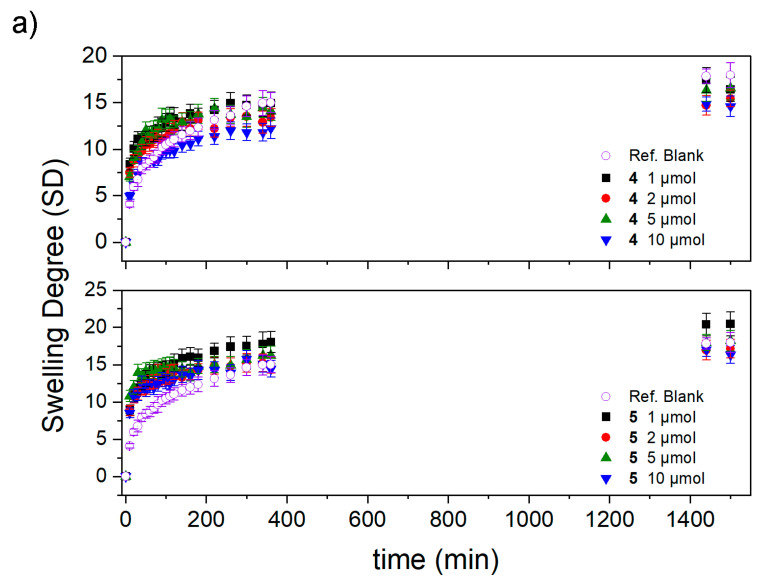

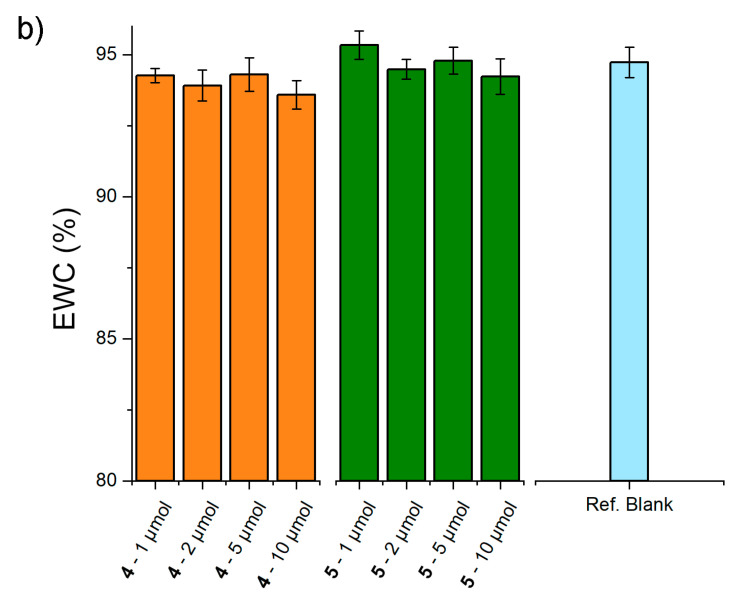
(**a**) Swelling behavior of gelatins treated with **4** and **5** at different concentrations in distilled H_2_O, compared to untreated gelatin (blank); (**b**) water content of hydrogels at equilibrium.

Gelatin treated with cross-linkers **4** and **5** was tested for its swelling behavior in water by gravimetric analysis. The swelling properties of gel are usually dependent upon several factors, including the pore size of the network, non-covalent interactions among the network (polymer chains and treating agents) and the solvent, and the chains’ mobility during the swelling process. The swelling degree (SD) and the equilibrium water content (EWC) for gels treated with **4** and **5** are reported in Figure 3. All of the hydrogel samples were prepared starting from the same quantity of freeze-dried specimens (50 mg).

The swelling behavior of samples treated with **4** is similar to untreated gelatin: a small increase in water absorption kinetic is observed for 1, 2, and 5 µmol, but not for 10 µmol. This behavior is much more pronounced in all the samples treated with **5**, with the first part of the SD curve noticeably higher than that of untreated gelatin. These observations are in accordance with the increased porosity of the tridimensional structure observed in the SEM micrographs. Equilibrium water content is a bit lower for samples treated with **4**, but in general, minimal differences are noticed.

#### 2.4.3. ATR-FTIR and ^1^H NMR Analyses

**Figure 4:**
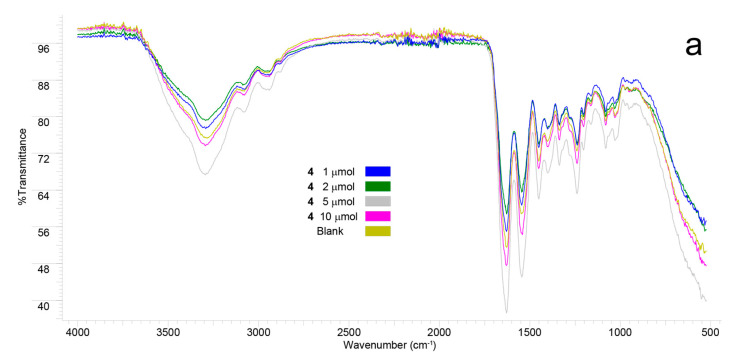

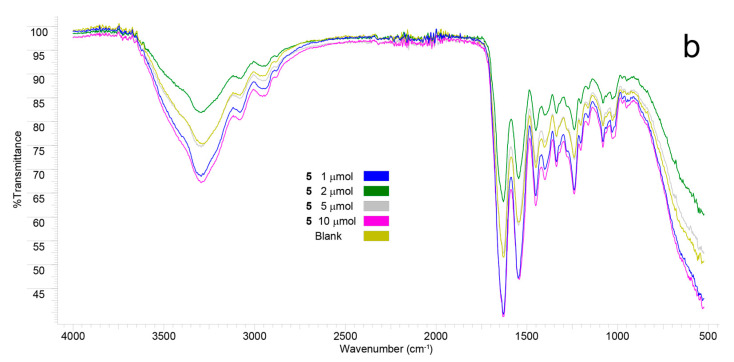
ATR-FTIR spectra of dry gelatins treated with **4** (**a**) and **5** (**b**).

The ATR-FTIR spectra of gelatin treated with **4** and **5** are reported in Figure 4. No significant differences can be detected at any concentration of tetrazoles in respect to untreated gelatin.

**Figure 5:**
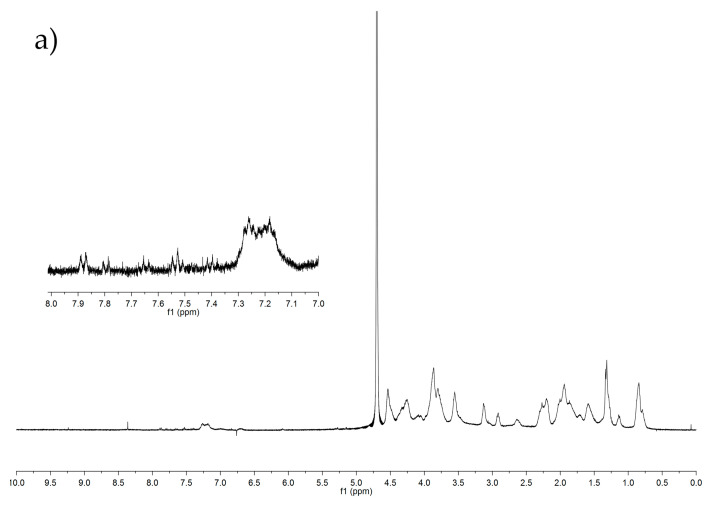

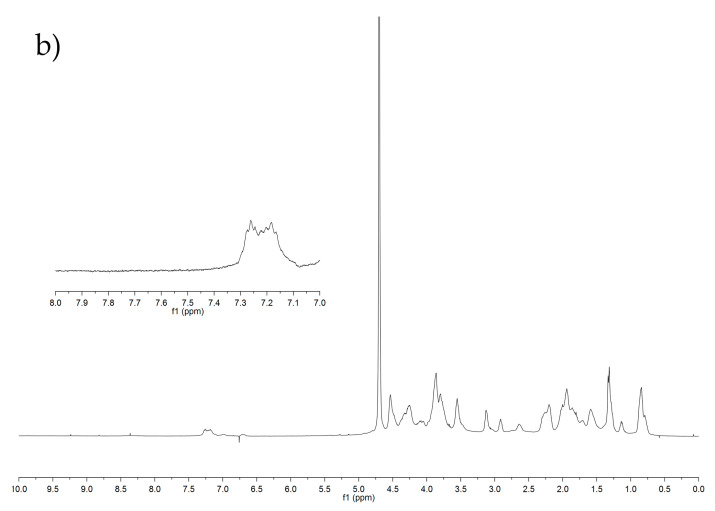
^1^H NMR (400 MHz, D_2_O) of gelatin treated with **5,** 5 µmol (**a**) compared to untreated gelatin (**b**).

Similarly, changes were almost never detected by ^1^H NMR analysis in the D_2_O of prepared specimens, which revealed the typical signal patterns of gelatin [71]. However, in samples treated with high concentrations of cross-linker **5**, the presence of a group of small signals was observed near that of gelatin’s tyrosine and phenylalanine resonances, attributable to the thiophene system of **5** (Figure 5a).

## 3. Conclusions

In summary, for the first time, the photoinduced gelatin cross-linking with homobi- and trifunctional tetrazoles via nitrilimine intermediates was proposed. Even if this chemistry has proven to be less effective than expected, which was also confirmed by the low degree of functionalization observed in the spectroscopic investigations, a significant increase in the thermal stability of gelatin hydrogels was achieved by using compounds **4** and especially **5**. The increased porosity and faster water absorption of treated samples deviate from the standard behavior of cross-linked biological matrices, which are normally less porous and less prone to water absorption compared to untreated starting materials. This study is of great importance for planning future applications of polyfunctional tetrazoles in photoinduced cross-linking of biological polymers, showing the necessity of planning the synthesis of cross-linkers endowed with multiple tetrazole photoactivable units.

## 4. Materials and Methods

### 4.1. General

All reagents and solvents were purchased from commercial sources (Merck Life Science S.r.l., Milan, Italy; Fluorochem Ltd., Hadfield, UK; and TCI Europe N.V., Zwijndrecht, Belgium) and used without further purification. Gelatin from porcine skin-Type A was used for the hydrogel preparation (Merk, catalog no. G2500). NMR spectra were recorded with a Bruker AVANCE III HD 400 MHz spectrometer (Bruker corp., Billerica, MA, USA). Two low-pressure mercury 15 W F15T8 UV-B lamps were simultaneously used for UV irradiation. The IR spectra were recorded with a Perkin Elmer Spectrum 100 FT-IR spectrometer equipped with a universal ATR sampling accessory (PerkinElmer Inc., Waltham, MA, USA). The scanning electron microscopy (SEM) analysis was performed with a Philips XL30 ESEM (FEI, Hillsboro, OR, USA). The gelatin was freeze-dried by a Christ alpha 1–2 freeze dryer (Christ, Osterode am Harz, Germany) at a temperature of −55 °C and at a pressure of 0.2–0.4 mBar. The UV λ_max_ was determined with a PerkinElmer Lambda 900 spectrophotometer (PerkinElmer Inc., Waltham, MA, USA). The melting points were measured with a Stanford Research Systems Optimelt apparatus (SRS, Sunnyvale, CA, USA).

### 4.2. Synthesis of Cross-Linking Agents

The synthetic procedures, complete characterizations, and ^1^H NMR, ^13^C NMR, and ATR-FTIR spectra of **1**, **2**, **3**, **4**, and **5** are reported in the Supplementary Materials.

### 4.3. Gelatin Cross-Linking

In a borosilicate flask (UV absorption below 300 nm), 100 mg of gelatin in 8 mL of DMSO were heated at 37 °C under magnetic stirring until complete dissolution (at least 8 h). After cooling to r.t., the cross-linking agent (1, 2, 5, and 10 µmol/100 mg of gelatin) was added and the mixture and stirred until homogeneity was achieved. The solution was then deoxygenated by N_2_ bubbling and irradiated at 310 nm (2 × 15 watt, at 20 cm) for 10 min, under vigorous stirring and an N_2_ atmosphere. THF (8 mL) was added to the solution, and the resulting suspension was centrifuged at 7500 rpm for 30 min before the supernatant was carefully removed. THF (8 mL) was added to the residue, and the mixture was vortexed (3000 rpm) for 2 min and centrifuged at 7500 rpm for 30 min. After careful removal of the supernatant, demineralized H_2_O (10 mL) was added, the suspension was vortexed (3000 rpm) for 2 min, centrifuged at 7500 rpm for 30 min, and the supernatant was carefully removed; this step was repeated twice. The obtained hydrogel was soaked in demineralized H_2_O overnight, recovered, and freeze-dried, yielding a weight of 60 to 80% that of the starting gelatin.

### 4.4. Thermal Stability

Freeze-dried, cross-linked gelatin specimens (ca 50 mg) and 4 control specimens worked in the same way as the treated samples were placed in tagged wells of a 24 multiwell plate, hydrated with PBS (4 mL, pH = 7.4, physiological conditions), and kept sealed at 37 °C. The specimens were filmed with a 720 p digital camera (video frames are presented in the Supplementary Materials) and periodically inspected.

### 4.5. SEM Iimaging

Scanning electron microscopy (SEM) analysis was performed working at 12 kV accelerating voltage and in low vacuum mode (0.8 Torr). Samples were dried, cut, fixed with conductive carbon tape to standard SEM stubs, and directly analyzed. Working in low vacuum conditions, no conductive coatings were applied, in order to preserve the original structure. Samples showed good stability under electron beam illumination at the operating conditions.

### 4.6. Swelling Studies

Dynamic swelling measurements were made by gravimetric measurements. Freeze-dried gelatin specimens (ca 50 mg) were soaked in distilled water at r.t. The swollen gels were periodically removed from water, blotted with filter paper, weighed on an analytical balance, and returned to the swelling medium until the equilibrium was reached.

The swelling degree (SD) was calculated from the following equation and reported as a function of time:

Swelling degree (SD, g·g^−1^) = (W_t_ − W_0_) · W_0_^−1^ (1)

where W_t_ is the weight of swelling hydrogel at different times and W_0_ is the dry weight of the gel.

The equilibrium water content (EWC), was calculated from the following equation:

EWC (%) = (W_e_ − W_0_) · We^−1^ · 100 (2)

where W_e_ is the swelling weight of the sample at equilibrium and W_0_ is the dry weight of the gel.

## Supplementary Materials

The following are available online at https://www.mdpi.com/article/10.3390/gels7030124/s1: procedures for the synthesis of cross-linkers **1**−**5**; ^1^H NMR, ^13^C NMR, and ATR-FTIR spectra of synthesized compounds; and video frames of the thermal stability test at 37 °C.
