# Supplementary material for: Photoinduced Porcine Gelatin Cross-Linking by Homobi- and Homotrifunctional Tetrazoles
Source: Gels. 2021 Aug 20;7(3):124. doi: 10.3390/gels7030124 (PMC8395868; doi:10.3390/gels7030124)

Supplementary Materials

Photo-induced porcine gelatin cross-linking by homobi- and homotrifunctional tetrazoles

Luca Vaghi[1], Mauro Monti[1], Marcello Marelli[2], Elisa Motto[3], Antonio Papagni[1] and Laura Cipolla[3]

[1] Dipartimento di Scienza dei Materiali, Università degli Studi di Milano-Bicocca, via R. Cozzi 55, 20125 Milano, Italy.
[2] Istituto di Scienze e Tecnologie Chimiche "Giulio Natta", CNR-SCITEC, Sede Fantoli, Via Fantoli 16/15, 20138 Milano, Italy.
[3] Dipartimento di Biotecnologie e Bioscienze, Università degli Studi di Milano-Bicocca, Piazza della Scienza 2, 20126 Milano, Italy.

Table of Contents

S2-S3. Procedures for the synthesis of 1-5.

S4-S11. ^{1}H NMR, ^{13}C NMR and ATR-FTIR spectra of synthesized compounds.

S12-S19. Video frames of thermal stability test at 37 °C.

Synthetic procedures

General information

All reagents and solvents were purchased from commercial sources (Fluorochem Co.; Tokyo Chemical Industry Co. and Aldrich Chemical Co.;) and used as received. Chromatographic purifications were performed using Merck 9385 silica gel, pore size 60 Å (230–400 mesh). Melting points were measured with a Stanford Research Systems Optimelt apparatus. IR spectra were recorded with a PerkinElmer Spectrum 100 FT-IR spectrometer equipped with universal ATR sampling accessory. ^{1}H and ^{13}C spectra were recorded with a Bruker AVANCE III HD 400 MHz spectrometer (^{1}H: 400 MHz, ^{13}C: 101 MHz), chemical shifts (δ) are expressed in parts per million (ppm), and coupling constants are given in Hz. Splitting patterns are indicated as follows: s = singlet, d = doublet, t = triplet, q = quartet, m = multiplet, br = broad. Elemental analyses were obtained with an Elementar vario MICRO cube instrument. UV λ_{max} were determined with a PerkinElmer Lambda 900 spectrophotometer.

*1,4-bis(2-phenyl-2H-tetrazol-5-yl)benzene (**1**).* A mixture of terephthalaldehyde (1.00 g, 7.5 mmol) and *p*-Toluenesulfonyl hydrazide (2.92 g, 15 mmol) in EtOH (35 mL) was heated at reflux under stirring for 5 h. After cooling to r.t., the mixture was poured into cold water. The precipitate formed was recovered by filtration and dried at 80°C overnight. The precipitate was then dissolved in 30 mL of pyridine to give solution A. In parallel, a solution of $NaNO_2$ (1.03 g, 15 mmol) in water (4 mL) was added dropwise to a cooled (0 °C) mixture of aniline (1.40 g, 15 mmol), concentrated HCl (37% in H_2O, 3.75 mL, 45 mmol), H_2O (5 mL) and EtOH (5 mL) to give solution B. Solution A was cooled with an ice bath and solution B was then slowly added. The mixture was stirred overnight at r.t.. The mixture was then poured into an aqueous HCl solution (200 mL, 3 M) and the precipitate was recovered by filtration. Crystallization (EtOAc) afforded **1** in a pure form (1.51 g, 55%). Pink solid; m.p.: 184-185 °C dec; ^{1}H NMR (400 MHz, $CDCl_3$) δ 8.44 (s, 4H), 8.26 – 8.22 (m, 4H), 7.64 – 7.58 (m, 4H), 7.57 – 7.50 (m, 2H). ^{13}C NMR (101 MHz, $CDCl_3$) δ 164.6, 149.7, 136.8, 136.1, 129.8, 129.7, 127.6, 123.8, 119.9. IR (ATR): 3191, 1596, 1560, 1492, 1471, 1450, 1425, 1361, 1322, 1300, 1277, 1213, 1187, 1165, 1093, 1053, 1011, 994, 954, 913, 854, 835, 811, 759, 739, 702, 677,571, 553 cm^{-1}; Anal. Calcd. for $C_{20}H_{14}N_8$: C, 65.56; H, 3.85; N, 30.58. Found: C, 65.67; H, 3.89; N, 30.45; UV (DMSO) λ_{max}, nm: 296.

*4,4'-bis(2-phenyl-2H-tetrazol-5-yl)-1,1'-biphenyl (**2**).* A mixture of (1,1'-biphenyl)-4,4'-dicarbaldehyde (2.50 g, 12 mmol) and *p*-Toluenesulfonyl hydrazide (4.43 g, 24 mmol) in EtOH (80 mL) was heated at reflux under stirring for 5 h. After cooling to r.t., the mixture was poured into cold water. The precipitate formed was recovered by filtration and dried at 80°C overnight. The precipitate was then dissolved in 90 mL of pyridine to give solution A. In parallel, a solution of $NaNO_2$ (1.65 g, 24 mmol) in water (6 mL)was added dropwise to a cooled (0 °C) mixture of aniline (2.22 g, 24 mmol), concentrated HCl (37% in H_2O, 6 mL, 72 mmol), H_2O (10 mL) and EtOH (10 mL) to give solution B. Solution A was cooled with an ice bath and solution B was then slowly added. The mixture was stirred overnight at r.t.. The mixture was then poured into an aqueous HCl solution (400 mL, 3 M) and the precipitate was recovered by filtration. Crystallization (MeOH) afforded **2** in a pure form (2.39 g, 45%). Pink solid; m.p.: 186-187 °C dec; ^{1}H NMR (400 MHz, DMSO-d_6) δ 8.34 (d, *J* = 8.0 Hz, 4H), 8.21 (d, *J* = 7.6 Hz, 4H), 8.07 (d, *J* = 8.0 Hz, 4H), 7.73 (t, *J* = 7.5 Hz, 4H), 7.67 (t, *J* = 7.2 Hz, 2H); IR (ATR): 3065, 3032, 2050, 1697, 1615, 1597, 1535, 1494, 1458, 1431, 1409, 1374, 1360, 1318, 1293, 1253, 1210, 1184, 1165, 1139, 1106, 1087, 1075, 1033, 1013, 992, 909, 863, 825, 748, 731, 715, 692, 675, 575 cm^{-1}; Anal. Calcd. for $C_{26}H_{18}N_8$: C, 70.58; H, 4.10; N, 25.32. Found: C, 70.70; H, 4.07; N, 25.21; UV (DMSO) λ_{max}, nm: 312.

*5-phenyl-2H-tetrazole (**6**).* A mixture of Benzonitrile (2.50 g, 24 mmol), sodium azide (1.73 g, 24 mmol) and zinc bromide (5.46 g, 24 mmol) in H_2O (50 mL) was heated at reflux under stirring for 24 h. The mixture was then cooled with an ice bath and acidified with concentrated HCl (37% H_2O) until pH ≈ 1. The mixture was extracted with EtOAc (3X 20 mL), the organic layer was dried (Na_2SO_4), and the solvent eliminated under reduced pressure. The crude was dissolved aqueous NaOH (200 mL, 0.25 M). The zinc salts formed were filtered off and the aqueous phase was acidified with aqueous HCl (400 mL, 3 M). The precipitate was recovered by filtration and dried at 80°C overnight to afford **6** in a pure form (2.64 g, 76 %). White solid; ^{1}H NMR (400 MHz, DMSO-d6) δ 8.08 – 8.04 (m, 2H), 7.64 – 7.57 (m, 3H); IR (ATR): 3130, 3056, 2980, 2905, 2834, 2794, 2763, 2684, 2649, 2601, 2543, 2480, 2450, 1898, 1857, 1824, 1765, 1713, 1609, 1563, 1485, 1466, 1439, 1409, 1288, 1256, 1084, 1055, 1035, 1015, 989, 956, 925, 840, 790, 784, 725, 703, 685 cm^{-1}. The physical and spectroscopic data corresponded to those reported in the literature [64].

*1,6-bis(5-phenyl-2H-tetrazol-2-yl)hexane (**3**).* 5-phenyl-2*H*-tetrazole **6** (0.76 g, 5.2 mmol) was dissolved in dry DMF (15 mL), under nitrogen atmosphere. K_2CO_3 (0.79 g, 5.7 mmol) was added in one portion and 1,6-dibromohexane (0.40 mL, 2.6 mmol) was added dropwise over a period of 10 min. The mixture was stirred at r.t. for 48 h. The mixture was then extracted with EtOAc (2X 20 mL), the organic layer was washed with brine (2X 20 mL), dried (Na_2SO_4) and the volatiles eliminated under reduced pressure. Flash column chromatography (SiO_2, CH_2Cl_2) afforded **3** in a pure form (0.51 g, 52%). White solid; m.p.: 210-211 °C dec; ^{1}H NMR (400 MHz, $CDCl_3$) δ 8.17 – 8.11 (m, 4H), 7.52 – 7.45 (m, 6H), 4.65 (t, *J* = 7.0 Hz, 4H), 2.13 – 2.02 (m, *J* = 7.2 Hz, 4H), 1.49 – 1.42 (m, 4H); ^{13}C NMR (101 MHz, $CDCl_3$) δ 165.1, 130.3, 128.9, 127.4, 126.8, 52.9, 29.1, 25.7; IR (ATR): 3067, 3034, 2947, 2872, 2863, 2165, 1981, 1962, 1895, 1822, 1767, 1716, 1653, 1610, 1585, 1528, 1463, 1449, 1397, 1365, 1352, 1339, 1306, 1286, 1251, 1206, 1177, 1131, 1103, 1070, 1042, 1030, 1000, 997, 921, 855, 787, 759, 729, 689, 617 cm^{-1}; Anal. Calcd. for $C_{20}H_{22}N_8$: C, 64.15; H, 5.92; N, 29.93. Found: C, 64.36; H, 5.89; N, 29.82; UV (DMSO) λ_{max}, nm: <265.

*4-(5-(thiophen-2-yl)-2H-tetrazol-2-yl)phenol (**7**).* A mixture of 2-Thiophenecarboxaldehyde (1.00 g, 8.9 mmol) and *p*-Toluenesulfonyl hydrazide (1.66 g, 8.9 mmol) in EtOH (25 mL) was heated at reflux under stirring for 5 h. After cooling to r.t., the mixture was poured into cold water. The precipitate formed was recovered by filtration and dried at 80°C overnight. The precipitate was then dissolved in 90 mL of pyridine to give solution A. In parallel, a solution of $NaNO_2$ (0.62 g, 8.9 mmol) in water (3 mL) added dropwise to a cooled (0 °C) mixture of 4-aminophenol (0.97 g, 8.92 mmol), concentrated HCl (37% in H_2O, 2 mL, 27 mmol), H_2O (5 mL) and EtOH (5 mL) to give solution B. Solution A was cooled with an ice bath and solution B was then slowly added. The mixture was stirred overnight at r.t.. The mixture was then poured into an aqueous HCl solution (200 mL, 3 M) and the precipitate was recovered by filtration. Flash column chromatography (SiO_2, CH_2Cl_2/EtOAc 95:5) afforded **2** in a pure form (2.39 g, 45%). Yellow solid; m.p. 160-161 °C dec; ^{1}H NMR (400 MHz, $CDCl_3$) δ 8.08 – 8.02 (m, 2H), 7.89 (dd, *J* = 3.6, 1.1 Hz, 1H), 7.49 (dd, *J* = 5.0, 1.1 Hz, 1H), 7.19 (dd, *J* = 5.0, 3.7 Hz, 1H), 7.04 – 6.99 (m, 2H), 5.38 (br. s, 1H); ^{13}C NMR (101 MHz, $CDCl_3$) δ 161.3, 156.8, 130.4, 128.9, 128.1, 128.1, 128.0, 121.7, 116.2; IR (ATR): 3177, 3108, 3093, 2983, 2952, 2925, 2847, 2701, 2632, 1883, 1811, 1743, 1680, 1644, 1618, 1600, 1575, 1569, 1516, 1474, 1410, 1368, 1340, 1328, 1281, 1254, 1230, 1221, 1201, 1187, 1168, 1124, 1106, 1089, 1074, 1053, 1018, 1005, 971, 939, 908, 851, 833, 747, 714, 705, 688, 669, 635, 628, 572 cm^{-1}

*1,4-bis(4-(5-(thiophen-2-yl)-2H-tetrazol-2-yl)phenoxy)butane (**4**).* **7** (0.40 g, 1.7 mmol) was dissolved in dry DMF (10 mL), under nitrogen atmosphere. K_2CO_3 (0.25 g, 1.8 mmol) was added in one portion and 1,4-dibromobutane (0.1 mL, 0.83 mmol) was added dropwise over a period of 10 min. The mixture was stirred r.t. for 72 h. The mixture was then extracted with EtOAc (2X 15 mL), the organic layer was washed with brine (2X 10 mL), dried (Na_2SO_4) and the volatiles eliminated under reduced pressure. Flash column chromatography (SiO_2, CH_2Cl_2) afforded **4** in a pure form (0.39 g, 87%). Yellow solid; m.p.: 190-191 °C dec; ^{1}H NMR (400 MHz, $CDCl_3$) δ 8.10 – 8.05 (m, 4H), 7.89 (dd, *J* = 3.7, 1.2 Hz, 2H), 7.49 (dd, *J* = 5.0, 1.2 Hz, 2H), 7.18 (dd, *J* = 5.0, 3.7 Hz, 2H), 7.06 – 7.01 (m, 4H), 4.08 (t, *J* = 6.0 Hz, 4H), 2.04 – 1.96 (m, 4H); ^{13}C NMR (101 MHz, $CDCl_3$) δ 161.1, 159.8, 130.3, 128.9, 128.1, 127.9, 67.3, 27.8; IR (ATR): 3075, 2957, 2939, 2869, 1980, 1888, 1808, 1732, 1609, 1600, 1570, 1517, 1471, 1437, 1431, 1406, 1397, 1376, 1351, 1302, 1276, 1258, 1225, 1204, 1182, 1114, 1066, 1042, 1015, 1001, 965, 852, 843, 827, 788, 748, 734, 704, 686, 682, 654, 638, 575 cm^{-1}; Anal. Calcd. for $C_{26}H_{22}N_8O_2S_2$: C, 57.55; H, 4.09; N, 20.65. Found: C, 57.22; H, 4.16; N, 20.71; UV (DMSO) λ_{max}, nm: 304.

*1,3,5-tris((4-(5-(thiophen-2-yl)-2H-tetrazol-2-yl)phenoxy)methyl)benzene (**5**).* **7** (0.40 g, 1.7 mmol) was dissolved in dry DMF (8 mL), under nitrogen atmosphere. K_2CO_3 (0.13 g, 0.94 mmol) was added in one portion and 1,3,5-tris(bromomethyl)benzene (0.83g, 0.23mmol) was added portionwise over a period of 10 min. The mixture was stirred r.t. for 72 h. The mixture was then extracted with EtOAc (2X 15 mL), the organic layer was washed with brine (2X 10 mL), dried (Na_2SO_4) and the volatiles eliminated under reduced pressure. Flash column chromatography (SiO_2, CH_2Cl_2) afforded **5** in a pure form (0.17 g, 87%). White solid; m.p. 192-193 °C dec; ^{1}H NMR (400 MHz, $CDCl_3$) δ 8.12 – 8.07 (m, 6H), 7.88 (dd, *J* = 3.6, 1.2 Hz, 3H), 7.55 (s, 3H), 7.49 (dd, *J* = 5.0, 1.1 Hz, 3H), 7.18 (dd, *J* = 5.0, 3.7 Hz, 3H), 7.16 – 7.11 (m, 6H), 5.22 (s, 6H); ^{13}C NMR (101 MHz, $CDCl_3$) δ 161.2, 159.4, 137.5, 130.6, 128.9, 128.1, 128.0, 126.1, 121.5, 115.6, 69.9; IR (ATR): 3107, 3082, 2925, 2854, 2558, 1885, 1805, 1729, 1680, 1608, 1596, 1572, 1509, 1475, 1457, 1440, 1409, 1373, 1300, 1247, 1225, 1203, 1175, 1112, 1066, 1043, 1012, 1002, 964, 893, 867, 850, 835, 807, 747, 701, 688, 673, 632, 587, 574 cm^{-1}; Anal. Calcd. for $C_{42}H_{30}N_{12}O_3S_3$: C, 59.56; H, 3.57; N, 19.85. Found: C, 59.22; H, 3.51; N, 19.94; UV (DMSO) λ_{max}, nm: 303.

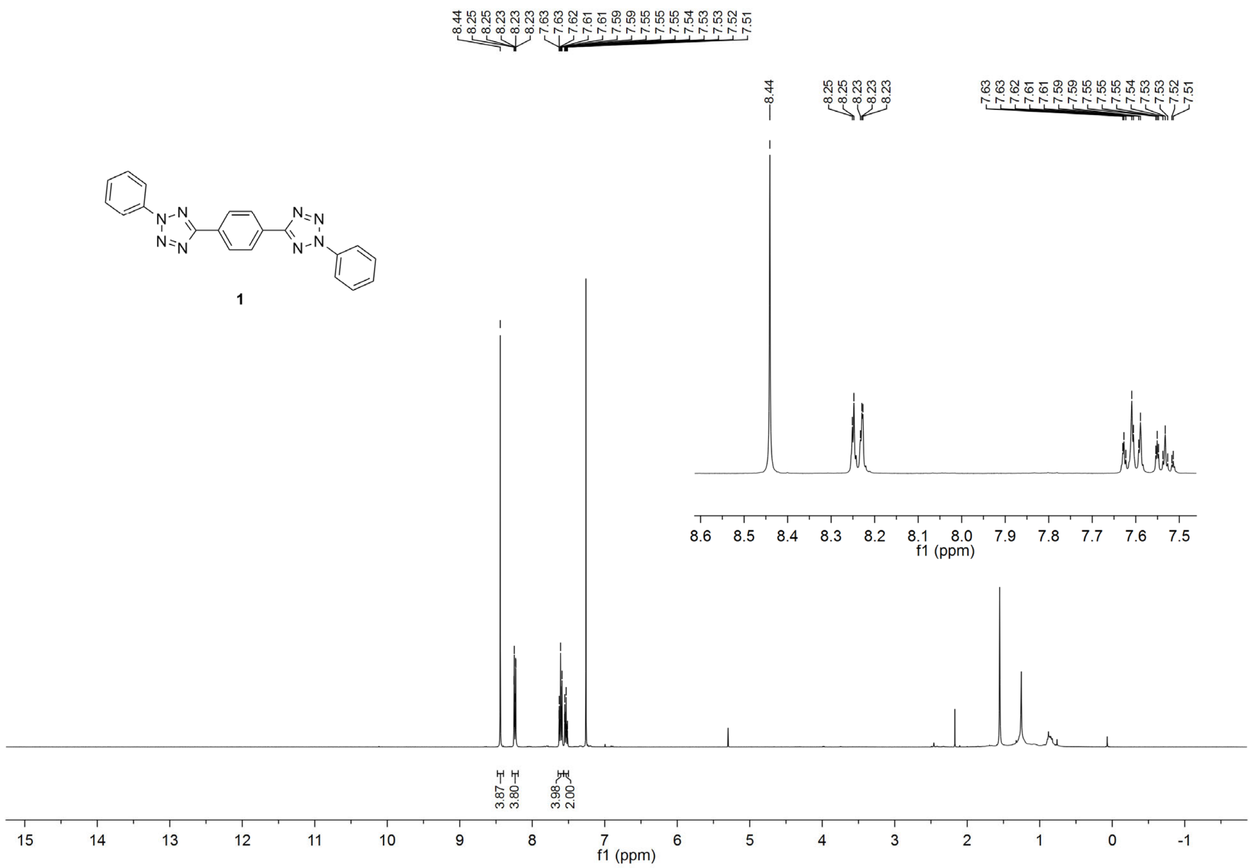
8.44
8.25
8.25
8.23
8.23
8.23
7.63
7.63
7.62
7.61
7.61
7.59
7.59
7.55
7.55
7.55
7.54
7.53
7.53
7.52
7.51
1
3.87
3.80
3.98
2.00
f1 (ppm)

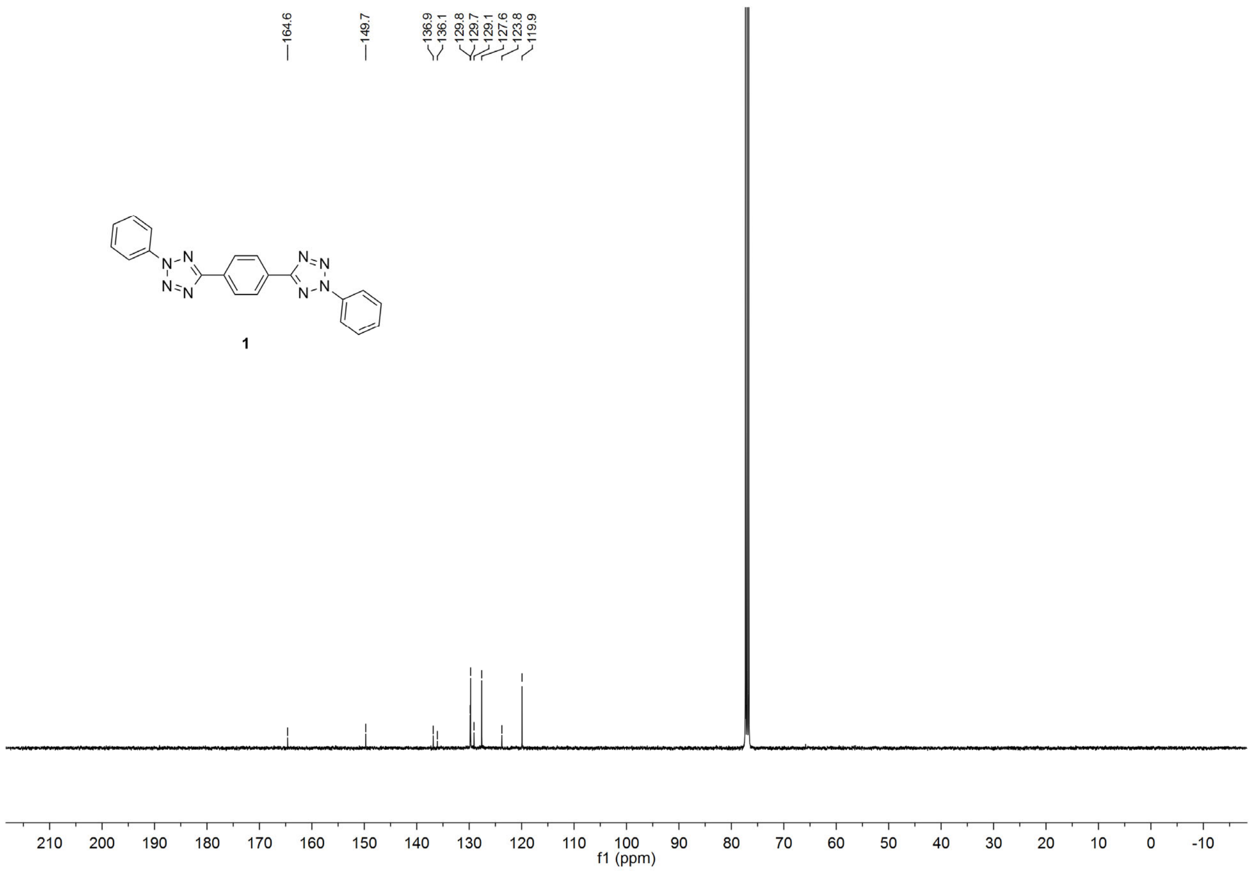
164.6
149.7
136.9
136.1
129.8
129.7
129.1
127.6
123.8
119.9
1
f1 (ppm)

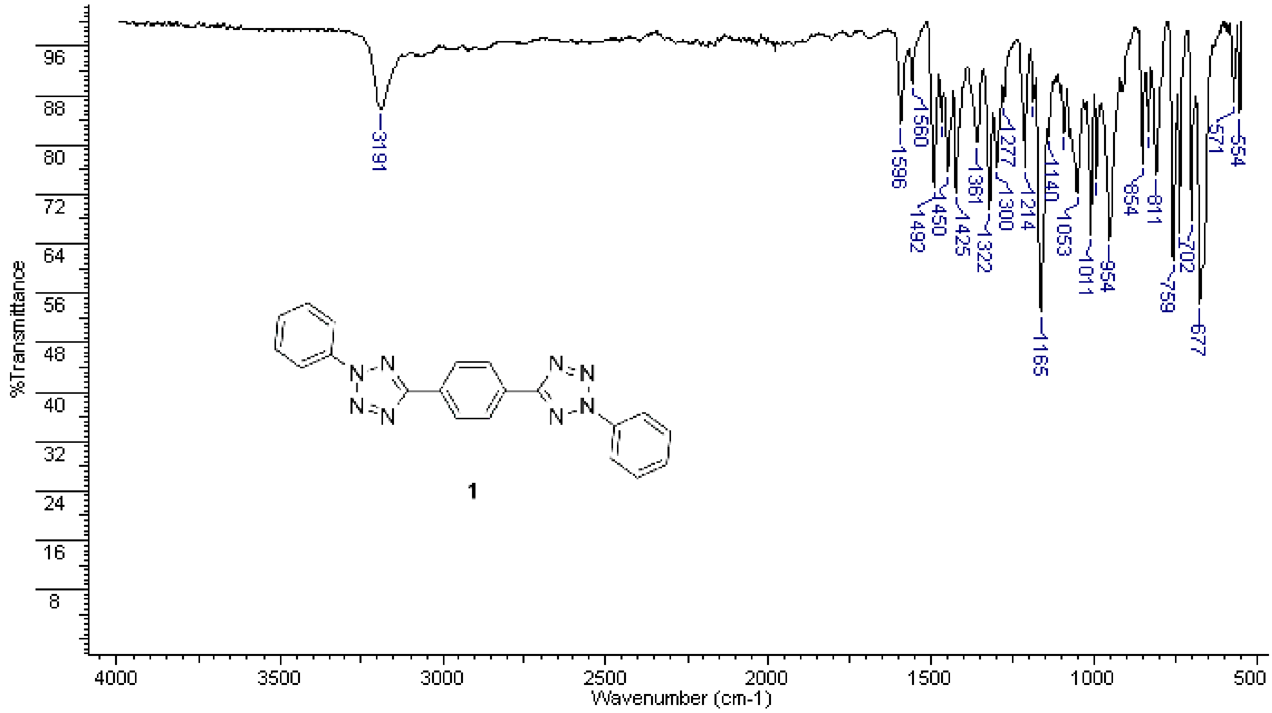

%Transmittance
Wavenumber (cm-1)
3191
1596
1560
1492
1450
1425
1361
1322
1300
1277
1214
1165
1140
1053
1011
954
854
811
759
702
677
571
554
1

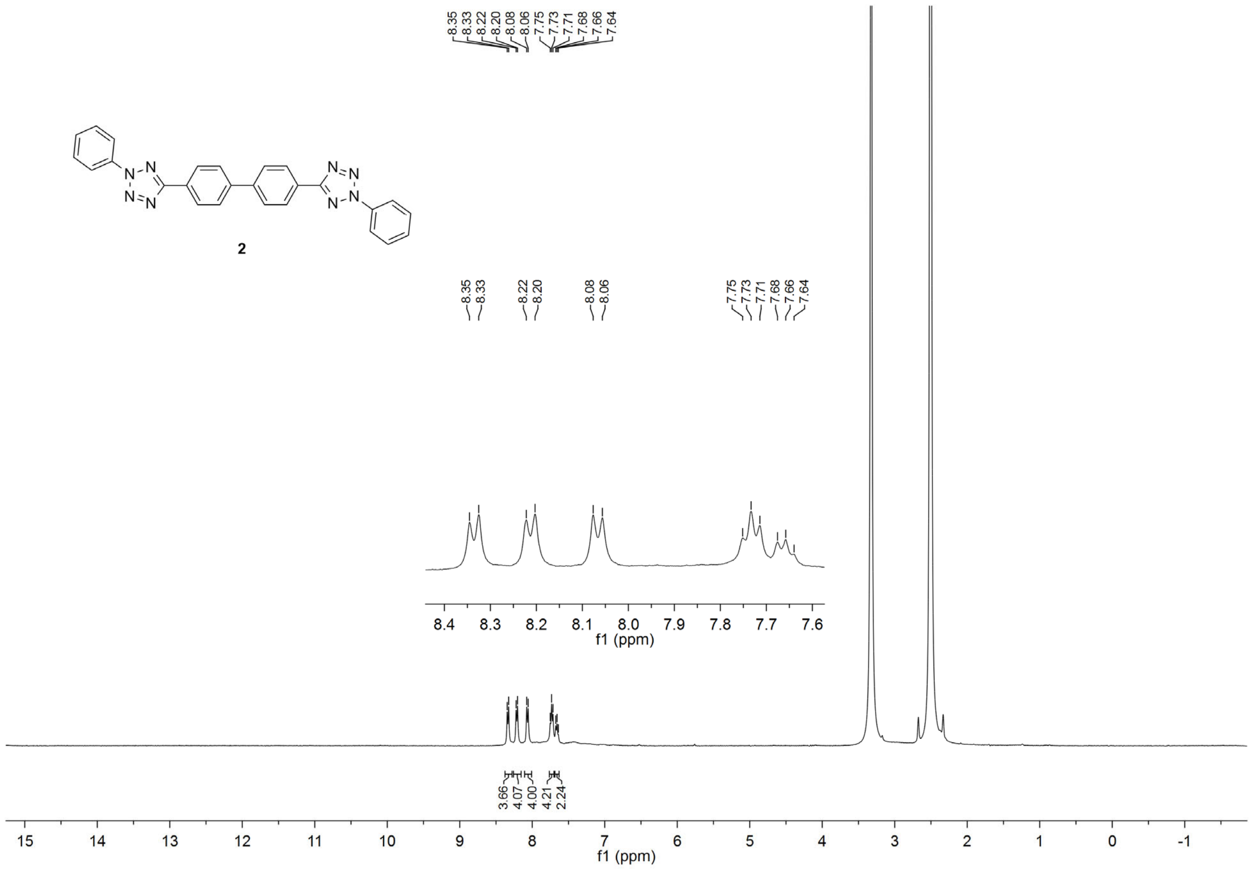

8.35
8.33
8.22
8.20
8.08
8.06
7.75
7.73
7.71
7.68
7.66
7.64
2
f1 (ppm)
3.66
4.07
4.00
4.21
2.24

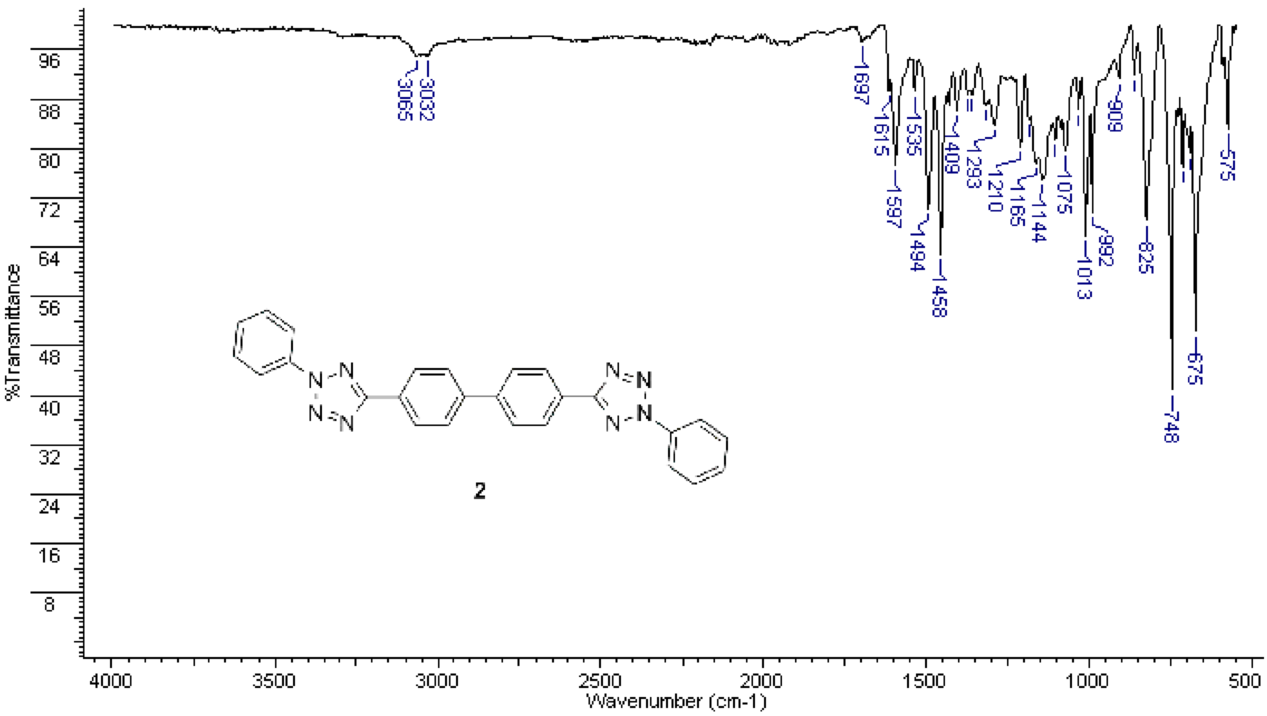

%Transmittance
Wavenumber (cm-1)
3065
3032
1697
1615
1597
1535
1494
1458
1409
1293
1210
1165
1144
1075
1013
992
909
825
748
675
575
2

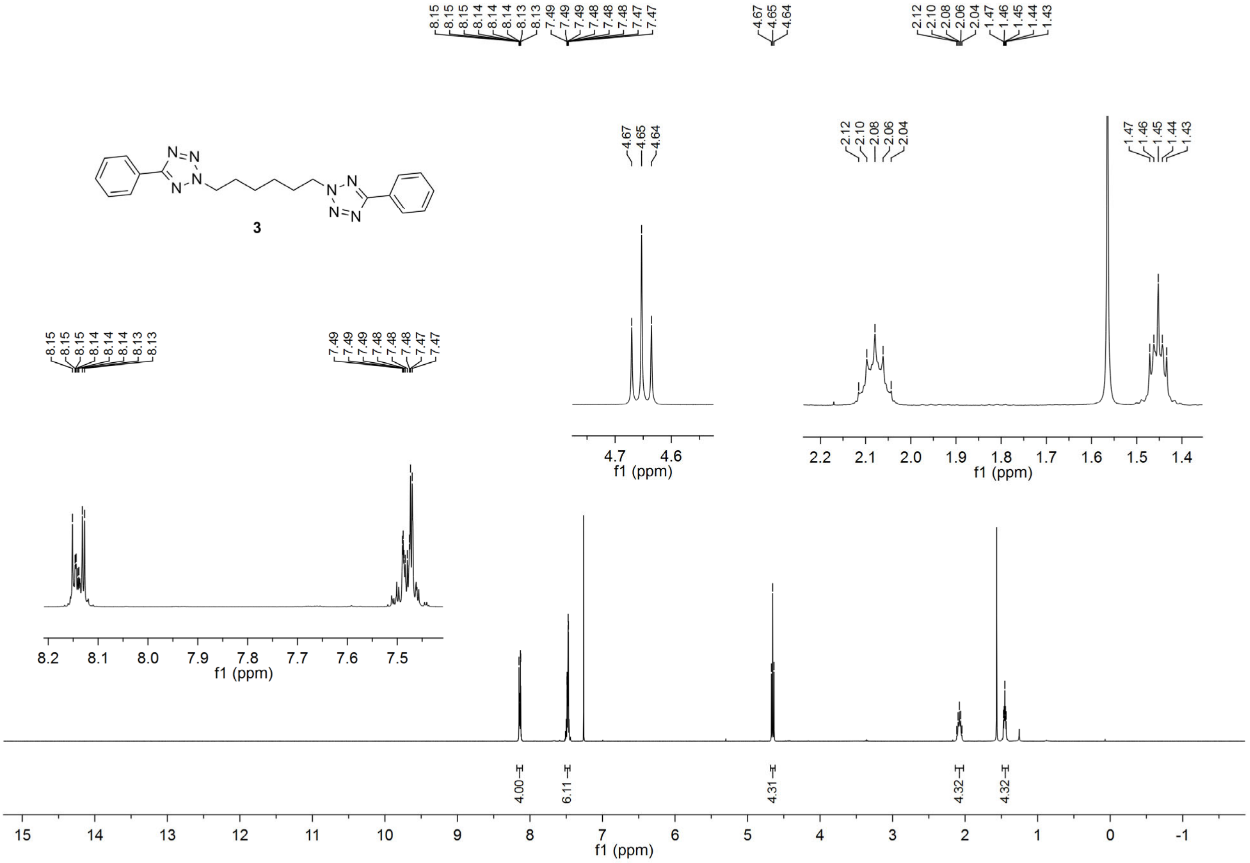

3
f1 (ppm)
4.00
6.11
4.31
4.32
4.32

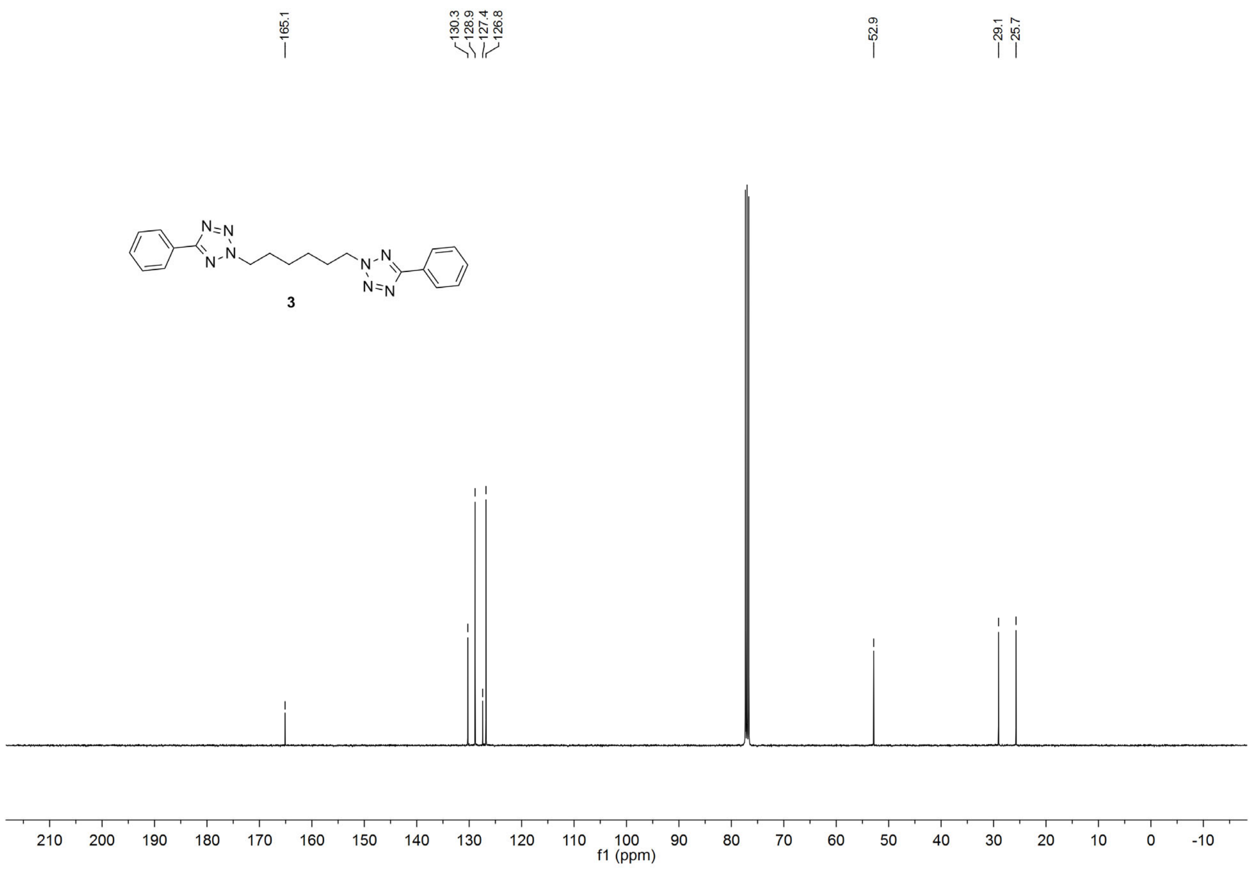

165.1
130.3
128.9
127.4
126.8
52.9
29.1
25.7
3
210 200 190 180 170 160 150 140 130 120 110 100 90 80 70 60 50 40 30 20 10 0 -10
f1 (ppm)

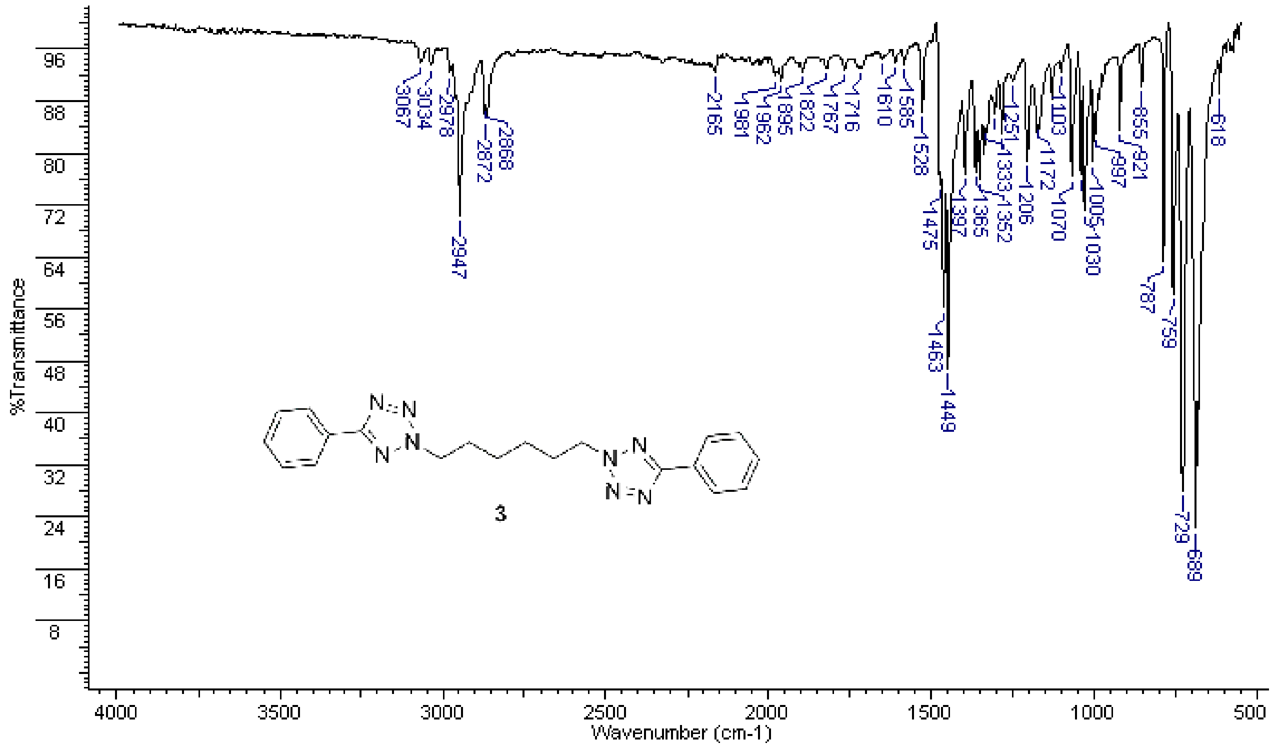

%Transmittance
96 88 80 72 64 56 48 40 32 24 16 8
3067
3034
2978
2947
2872
2868
2165
1981
1962
1895
1822
1767
1716
1610
1585
1528
1475
1463
1449
1397
1365
1352
1333
1251
1206
1172
1103
1070
1030
1005
997
921
855
787
759
729
689
618
3
4000 3500 3000 2500 2000 1500 1000 500
Wavenumber (cm-1)

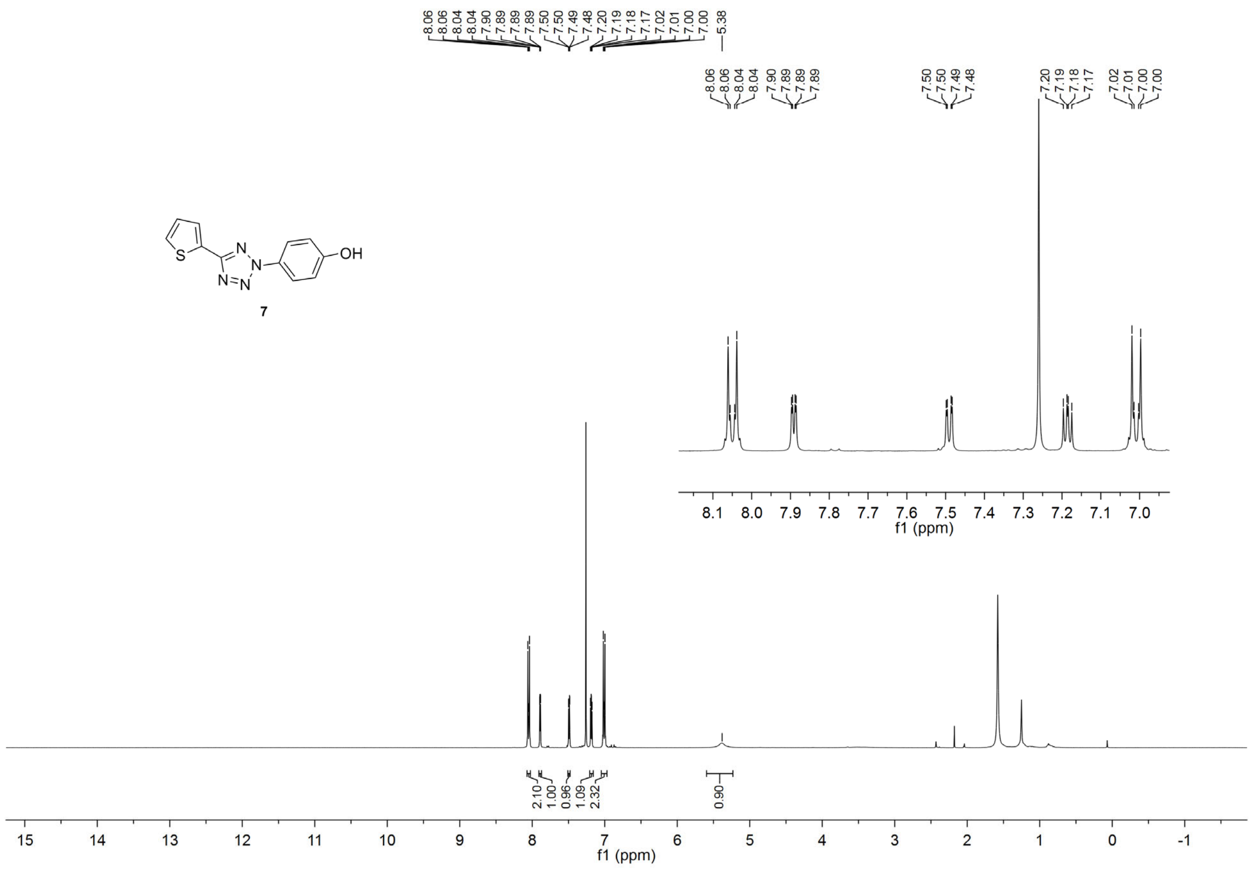
8.06
8.06
8.04
8.04
7.90
7.89
7.89
7.89
7.50
7.50
7.49
7.48
7.20
7.19
7.18
7.17
7.02
7.01
7.00
7.00
5.38
N
N
N
N
S
OH
7
2.10
1.00
0.96
1.09
2.32
0.90
f1 (ppm)

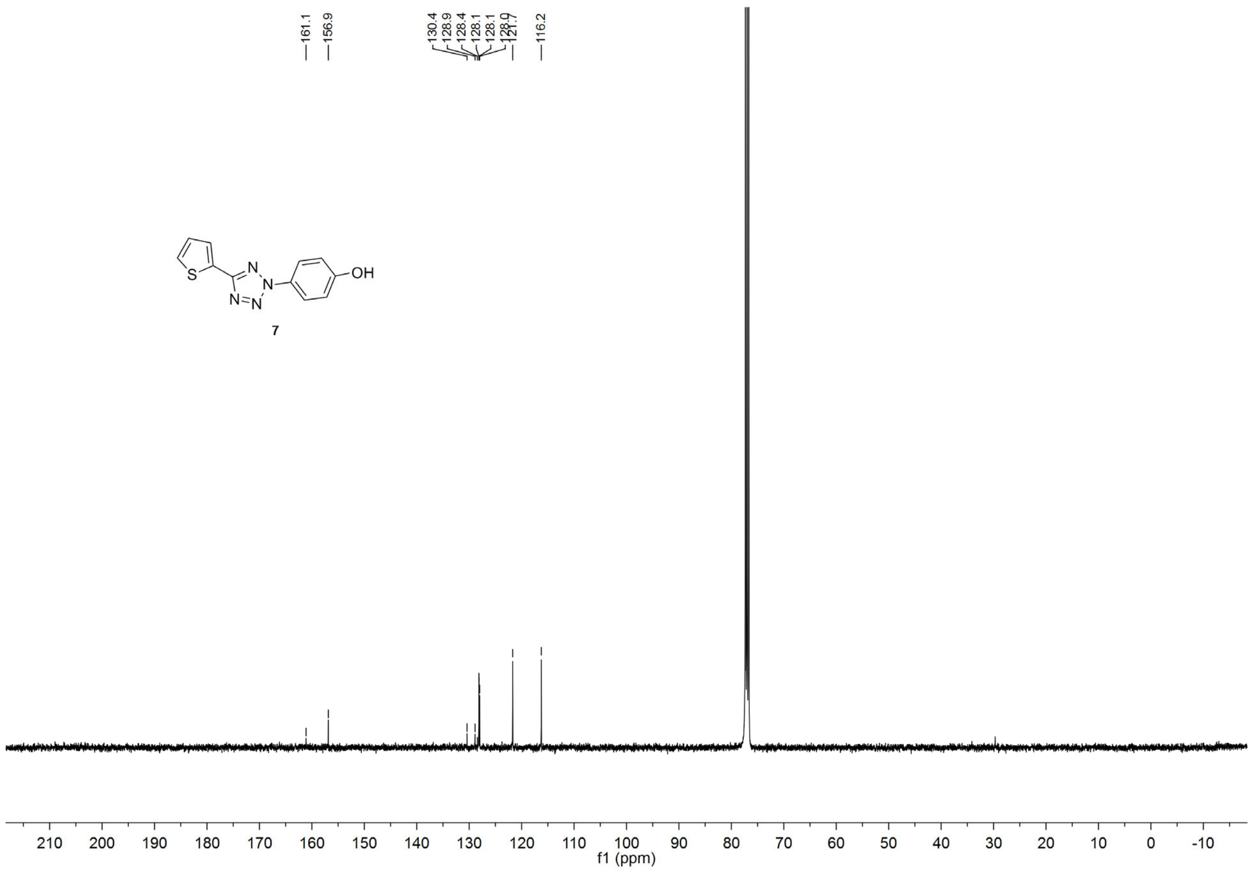
161.1
156.9
130.4
128.9
128.4
128.1
128.1
128.0
121.7
116.2
N
N
N
N
S
OH
7
f1 (ppm)

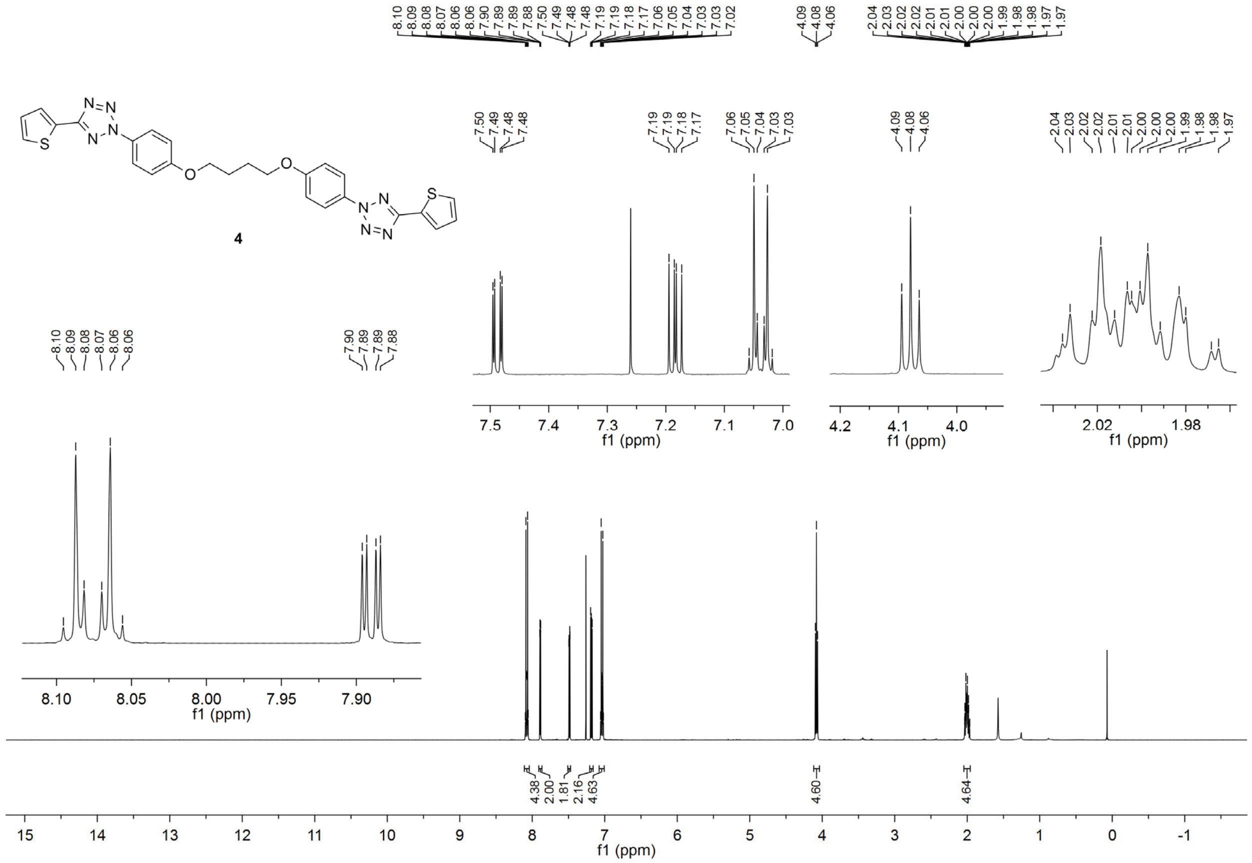

8.10 8.09 8.08 8.07 8.06 8.06 7.90 7.89 7.89 7.88 7.50 7.49 7.48 7.48 7.19 7.19 7.18 7.17 7.06 7.05 7.04 7.03 7.03 7.02
4.09 4.08 4.06
2.04 2.03 2.02 2.02 2.01 2.01 2.00 2.00 2.00 1.99 1.98 1.98 1.97
4
4.38 2.00 1.81 2.16 4.63
4.60
4.64
f1 (ppm)

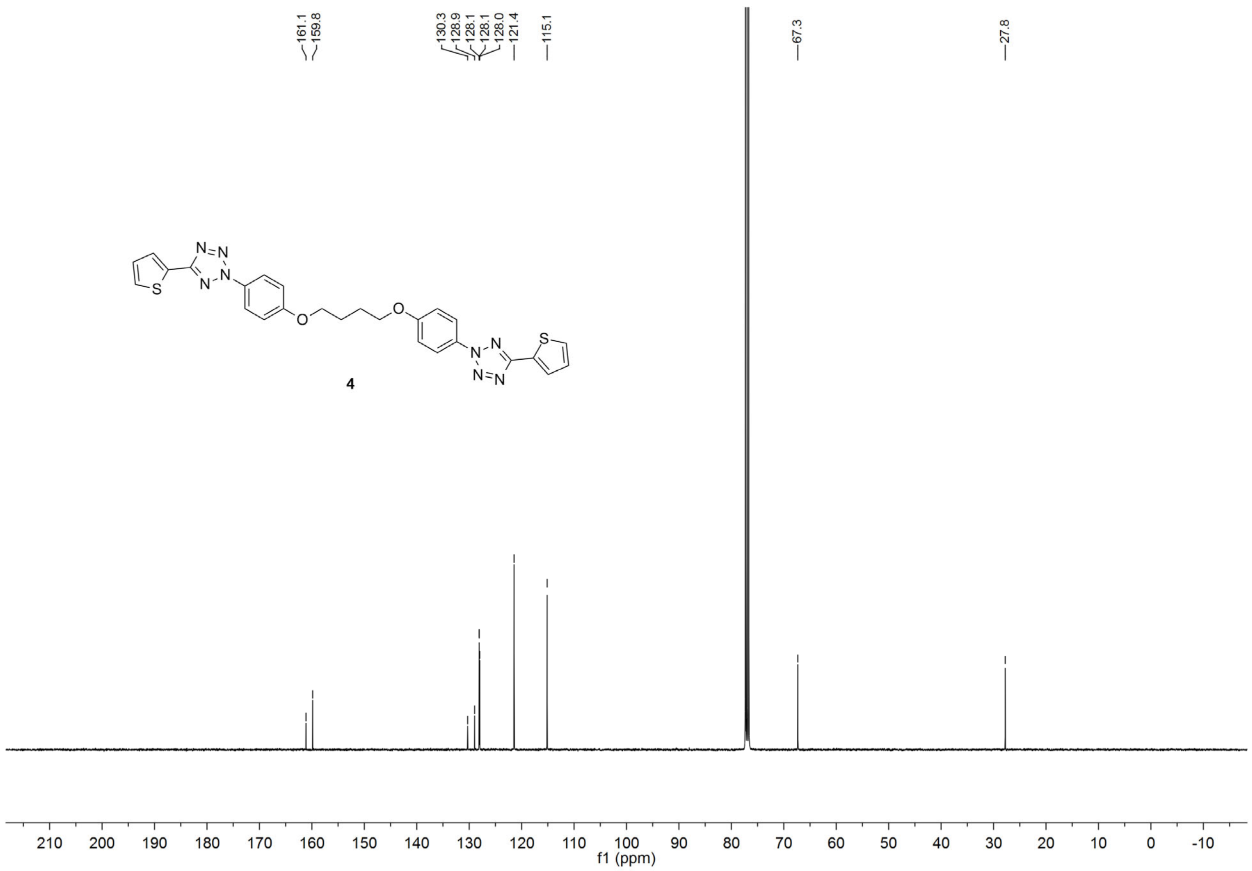

161.1 159.8
130.3 128.9 128.1 128.1 128.0 121.4
115.1
67.3
27.8
4
f1 (ppm)

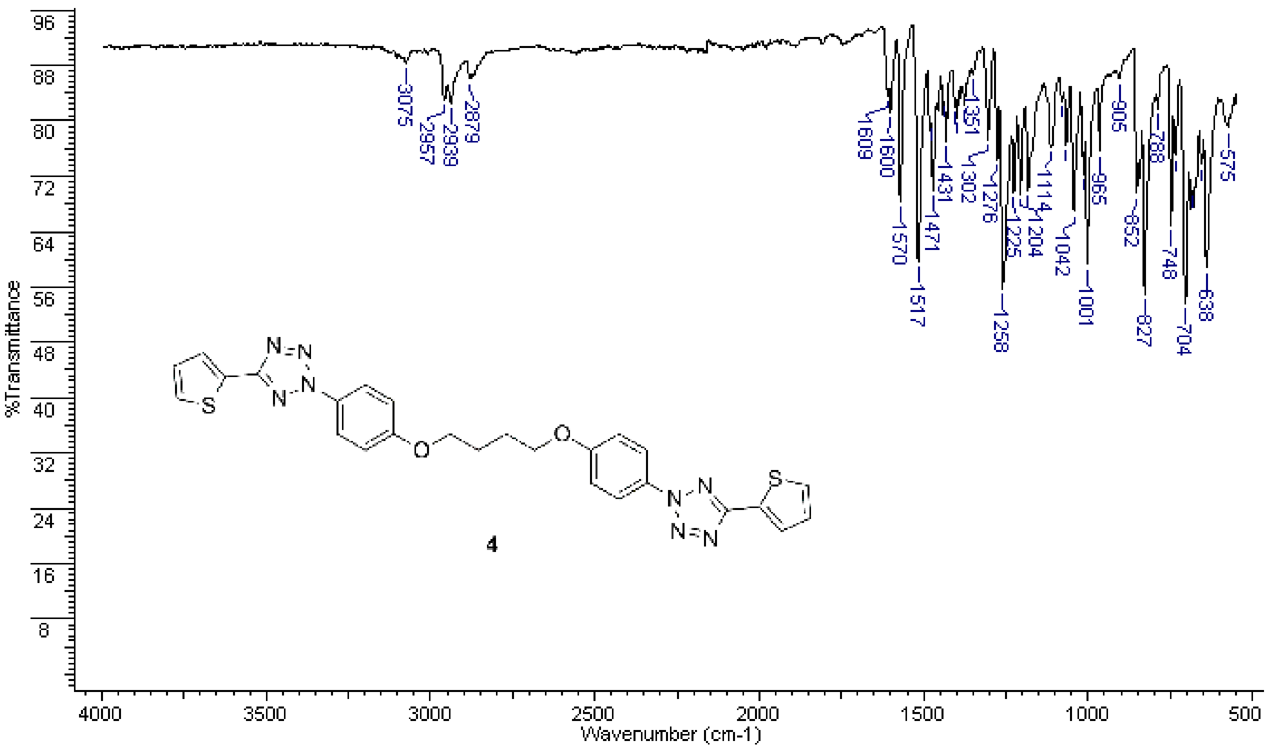

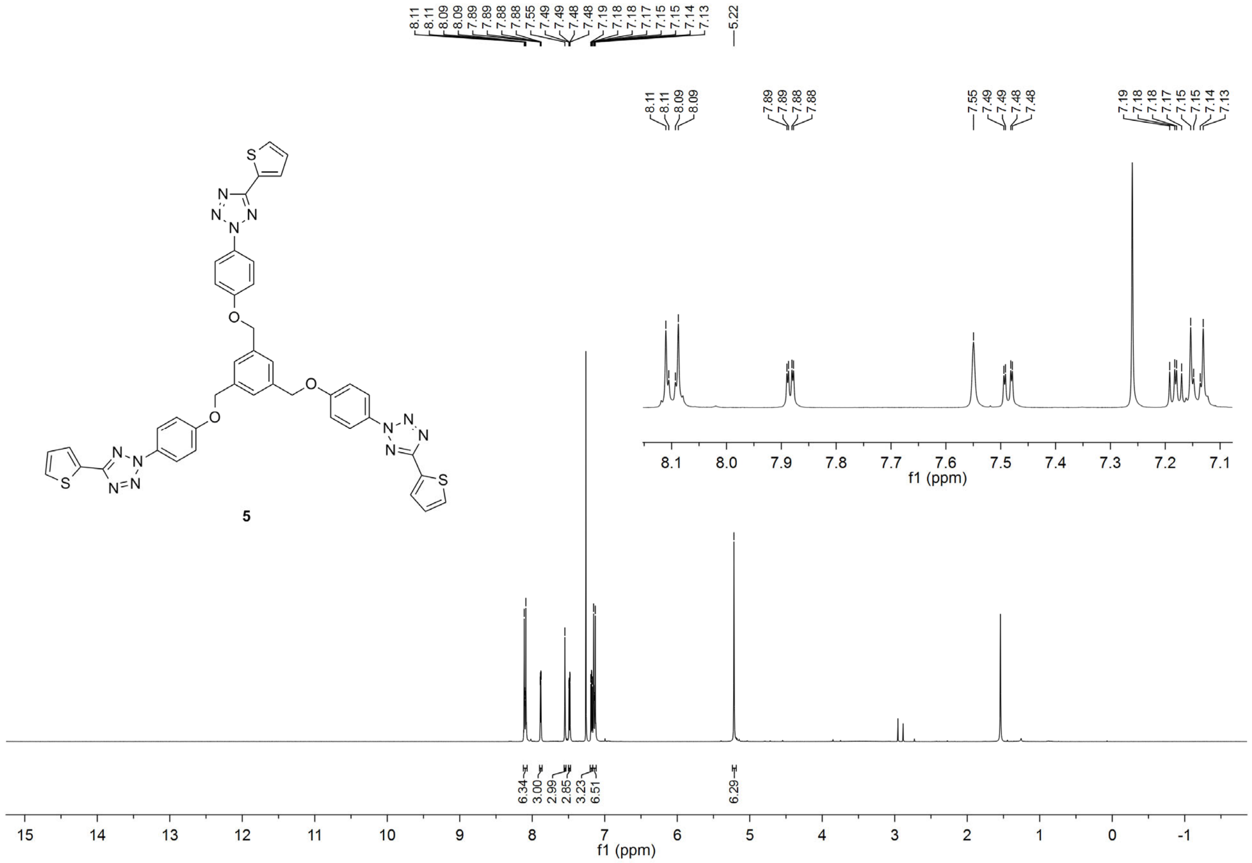

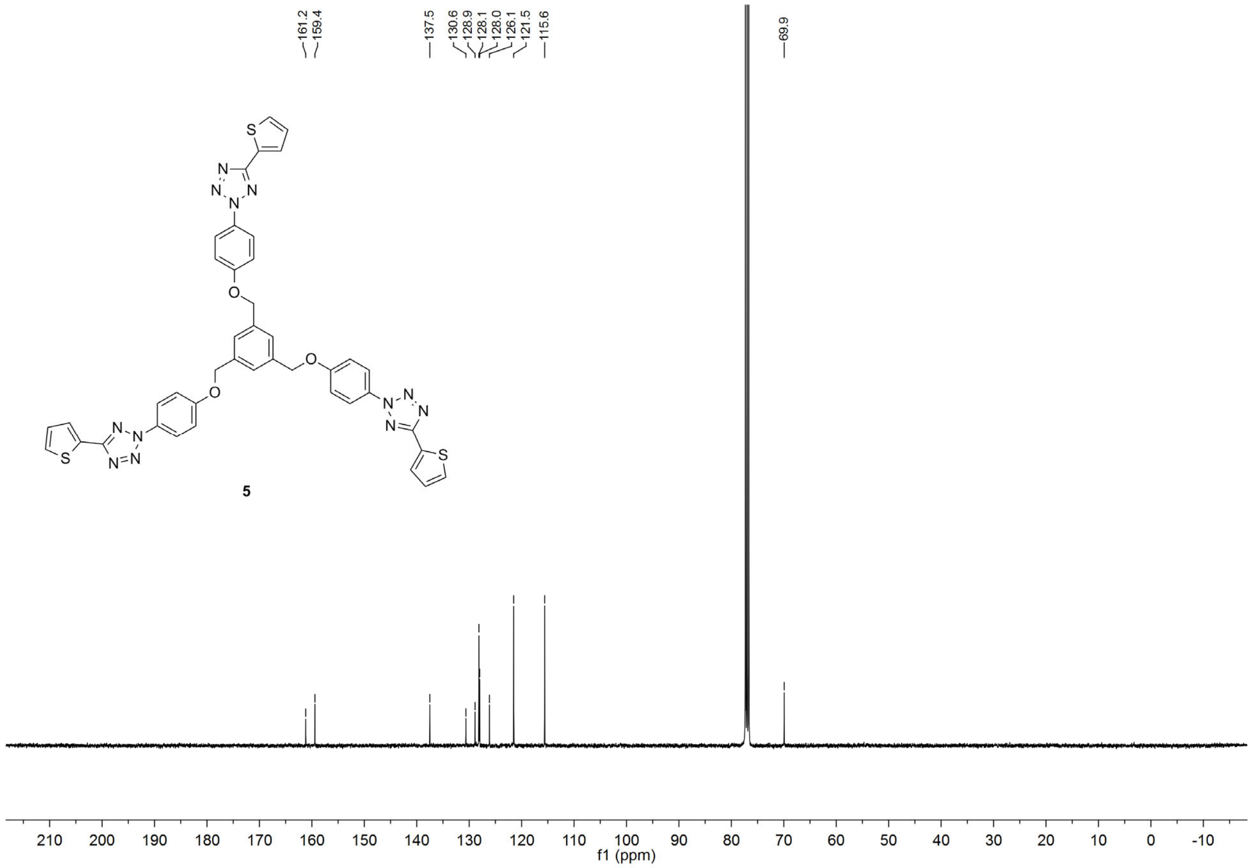
161.2
159.4
137.5
130.6
128.9
128.1
128.0
126.1
121.5
115.6
69.9
5
210
200
190
180
170
160
150
140
130
120
110
100
90
80
70
60
50
40
30
20
10
0
-10
f1 (ppm)

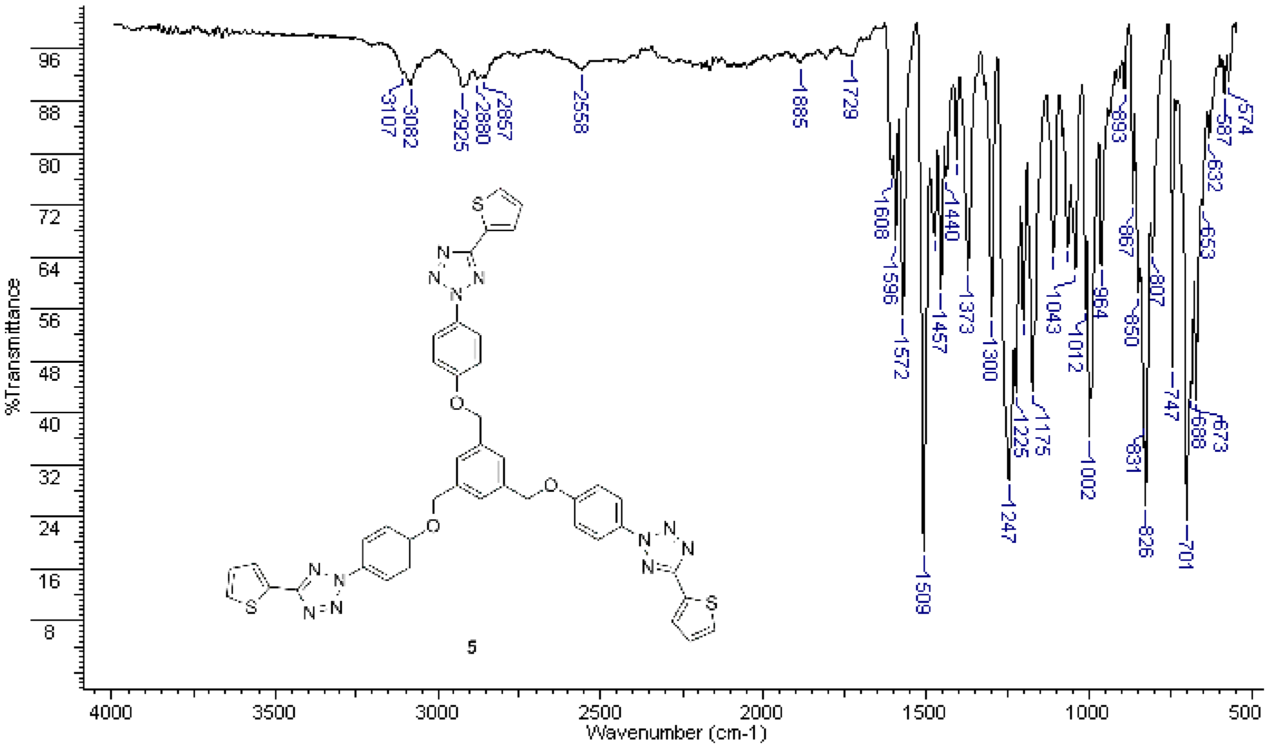
%Transmittance
Wavenumber (cm-1)
3107
3082
2925
2880
2857
2558
1885
1729
1608
1596
1572
1509
1457
1440
1373
1300
1247
1225
1175
1043
1012
1002
964
893
867
850
831
826
807
747
701
688
673
653
632
587
574
5

Legend:

Blank	Blank	**5**<br>10 μmol	**5**<br>5 μmol	**5**<br>2 μmol	**5**<br>1 μmol
Blank	Blank	**3**<br>10 μmol	**3**<br>5 μmol	**3**<br>2 μmol	**3**<br>1 μmol
**4**<br>10 μmol	**4**<br>5 μmol	**2**<br>10 μmol	**2**<br>5 μmol	**2**<br>2 μmol	**2**<br>1 μmol
**4**<br>2 μmol	**4**<br>1 μmol	**1**<br>10 μmol	**1**<br>5 μmol	**1**<br>2 μmol	**1**<br>1 μmol

t_0:

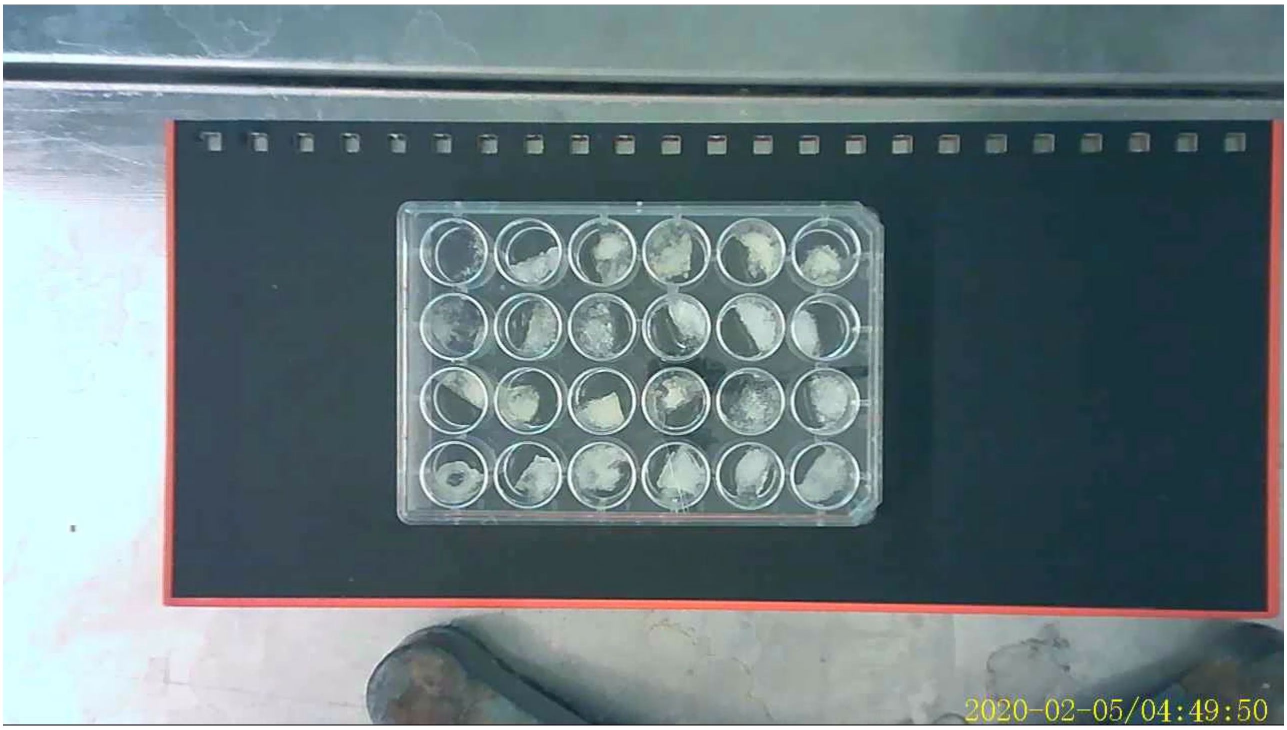

15 min:

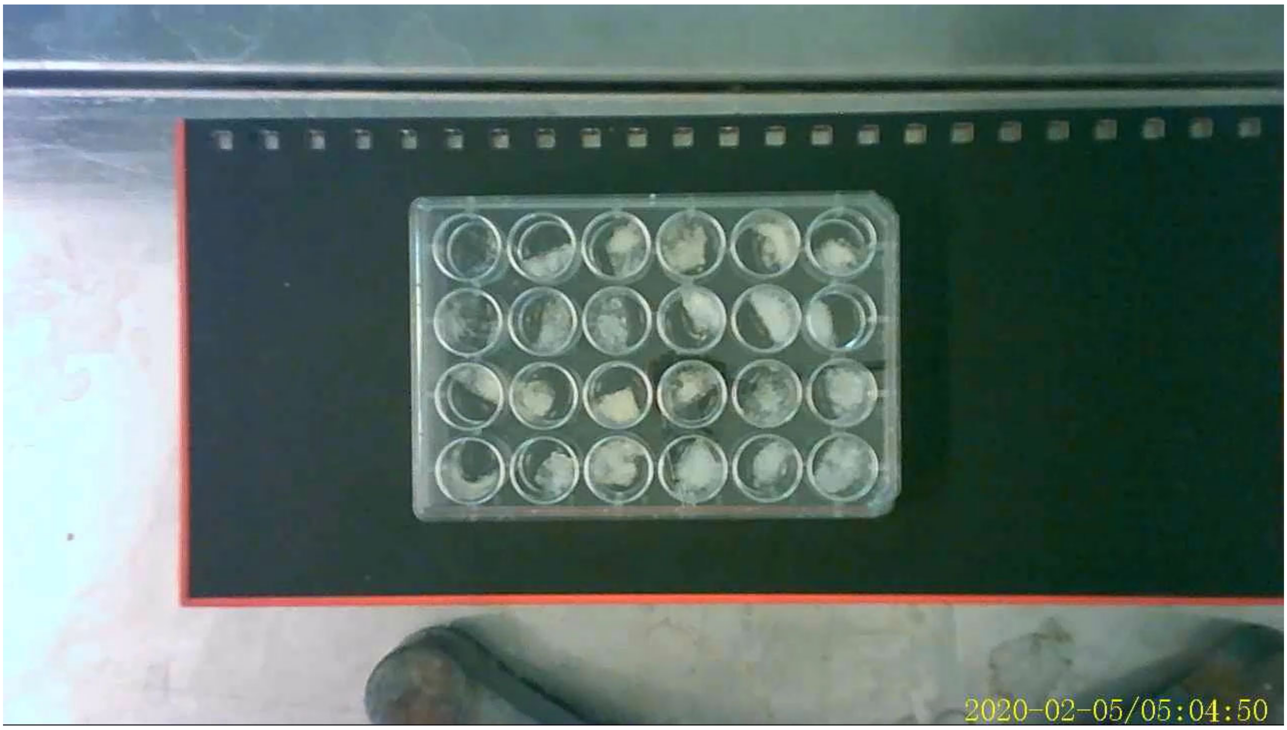

30 min:

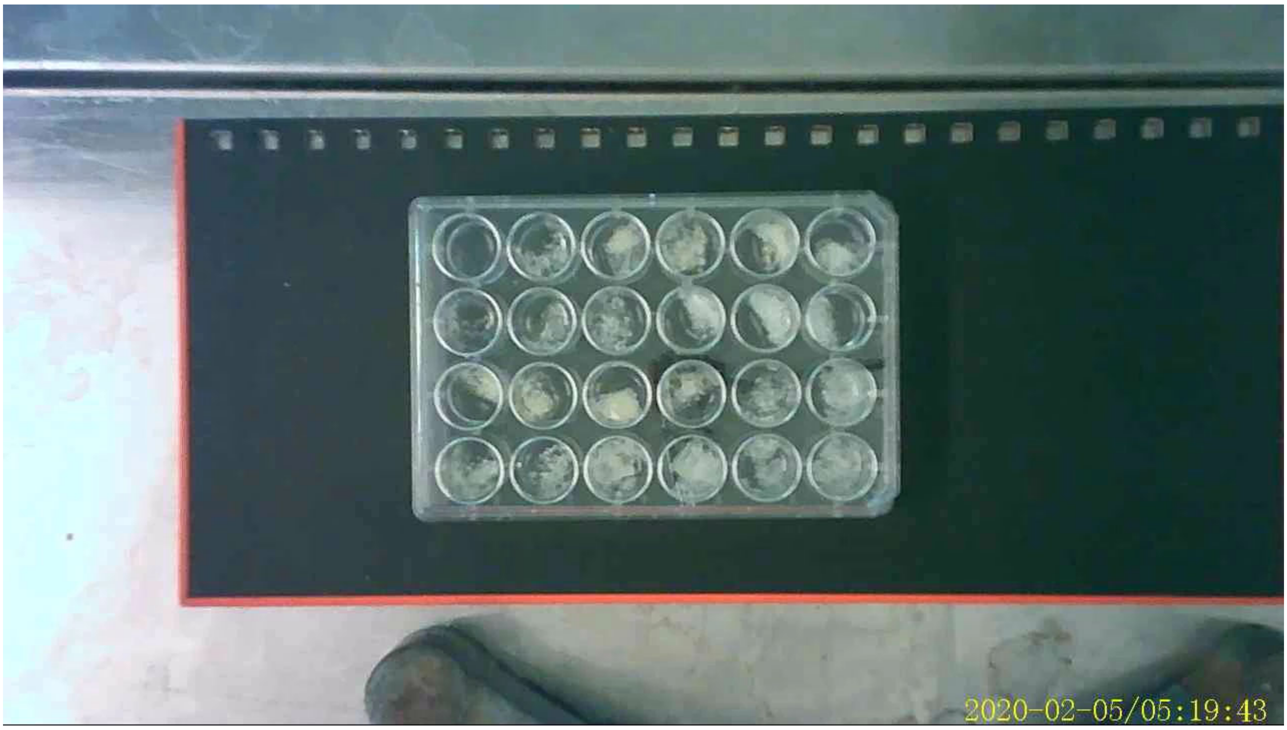

45 min:

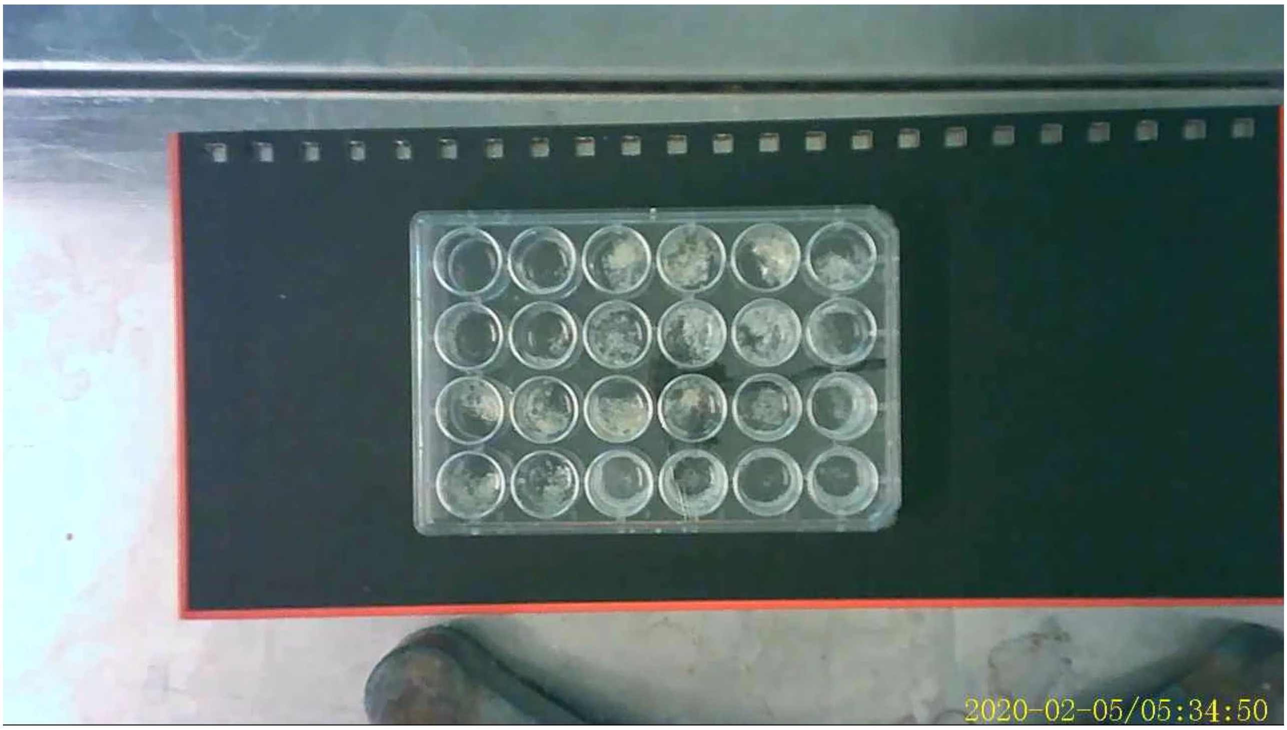

60 min:

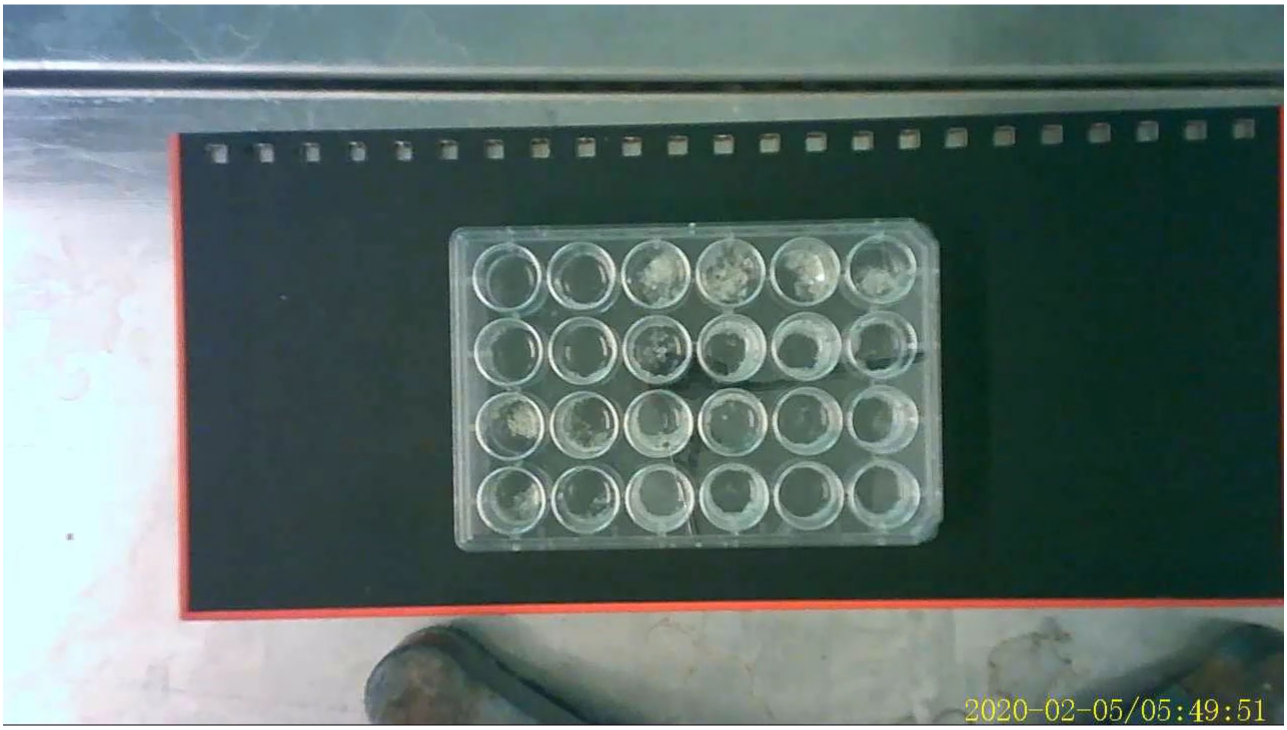

75 min:

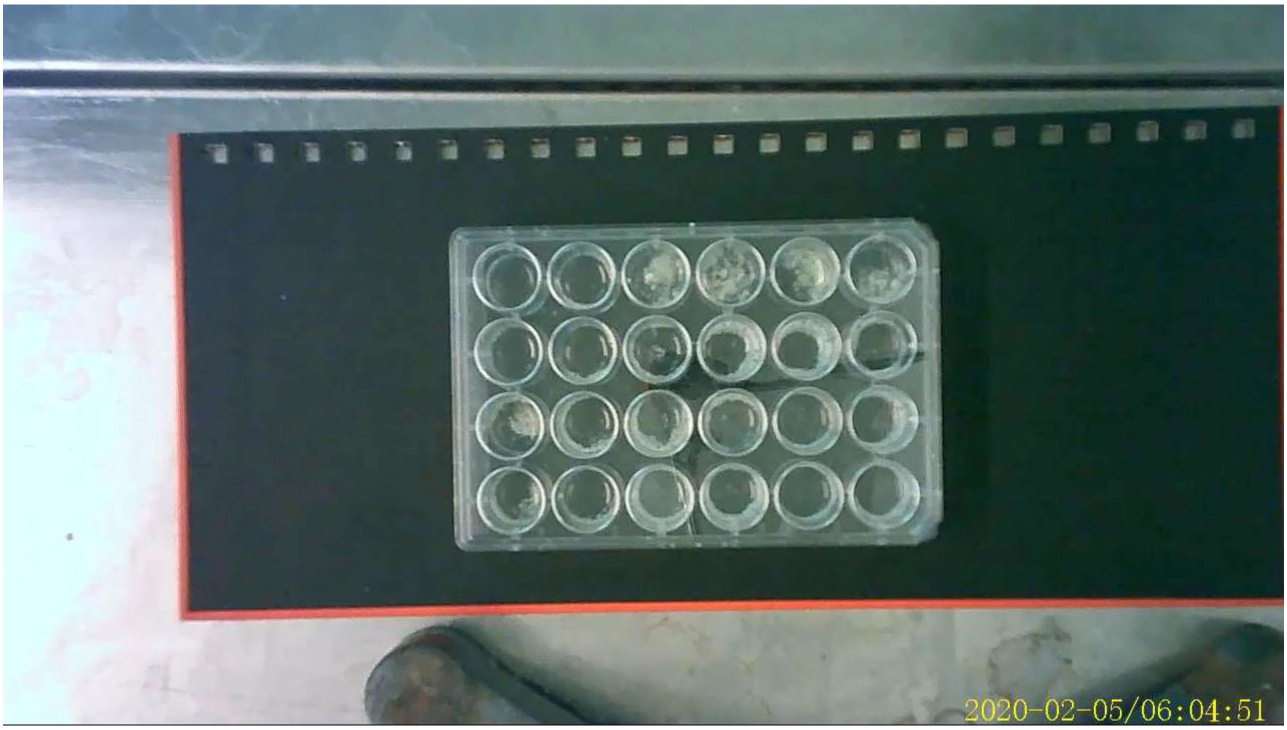

90 min:

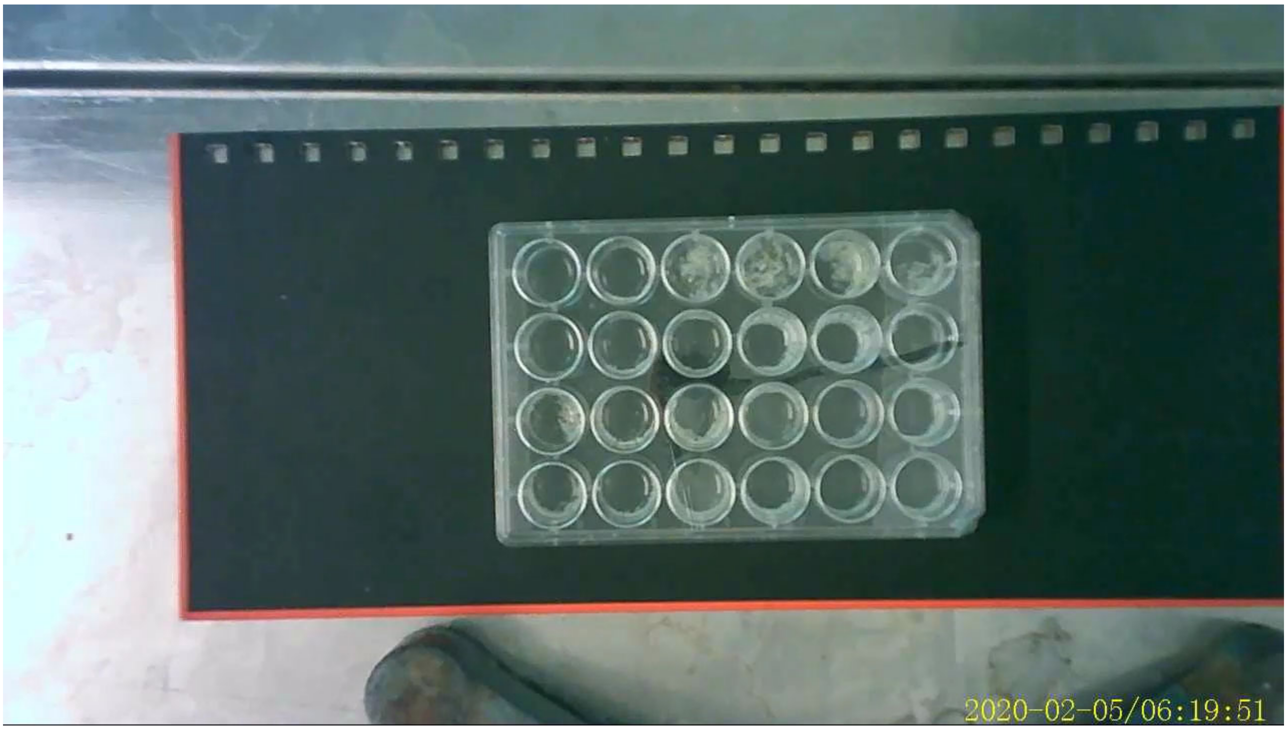

105 min:

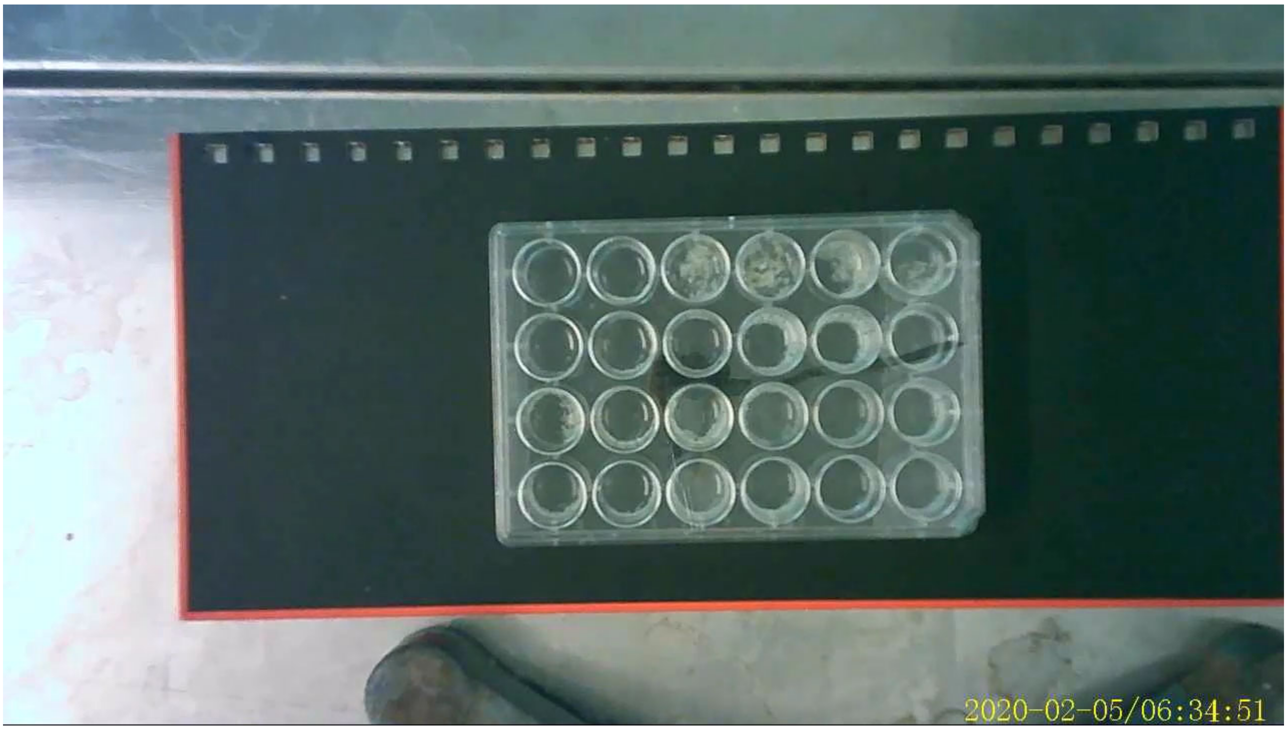

120 min:

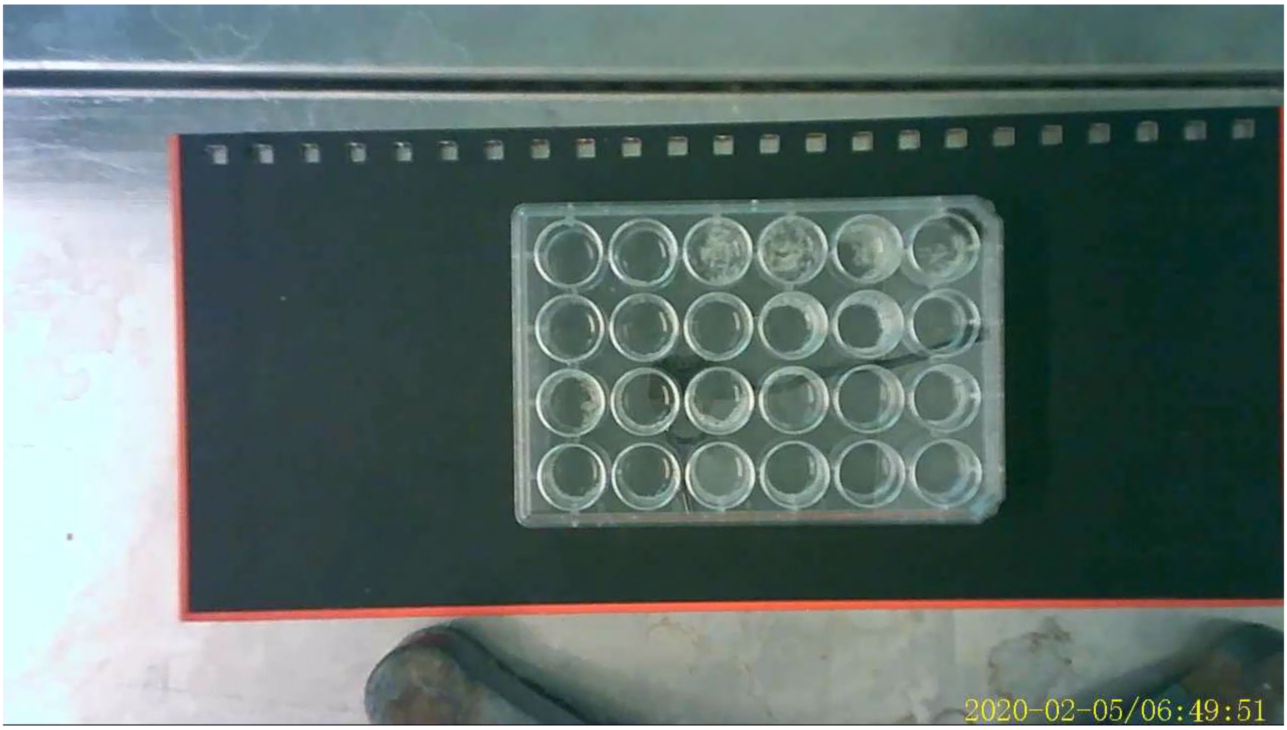

135 min:

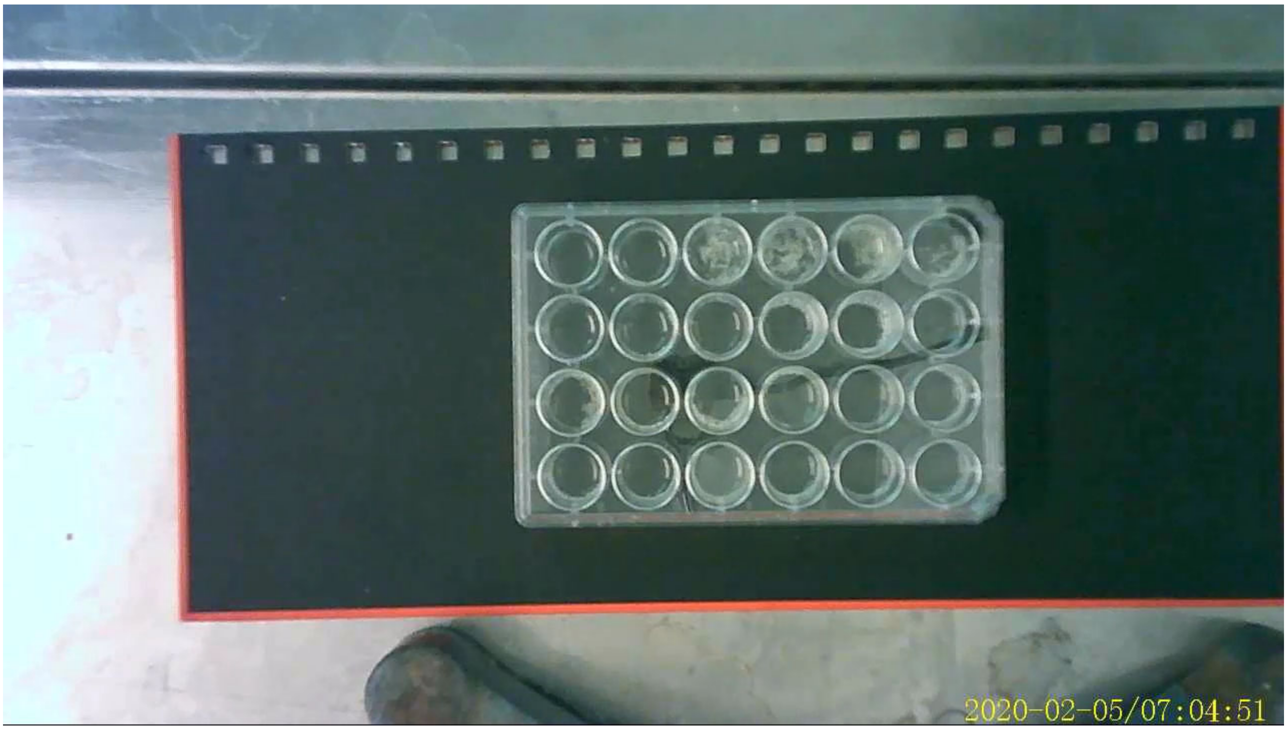

150 min:

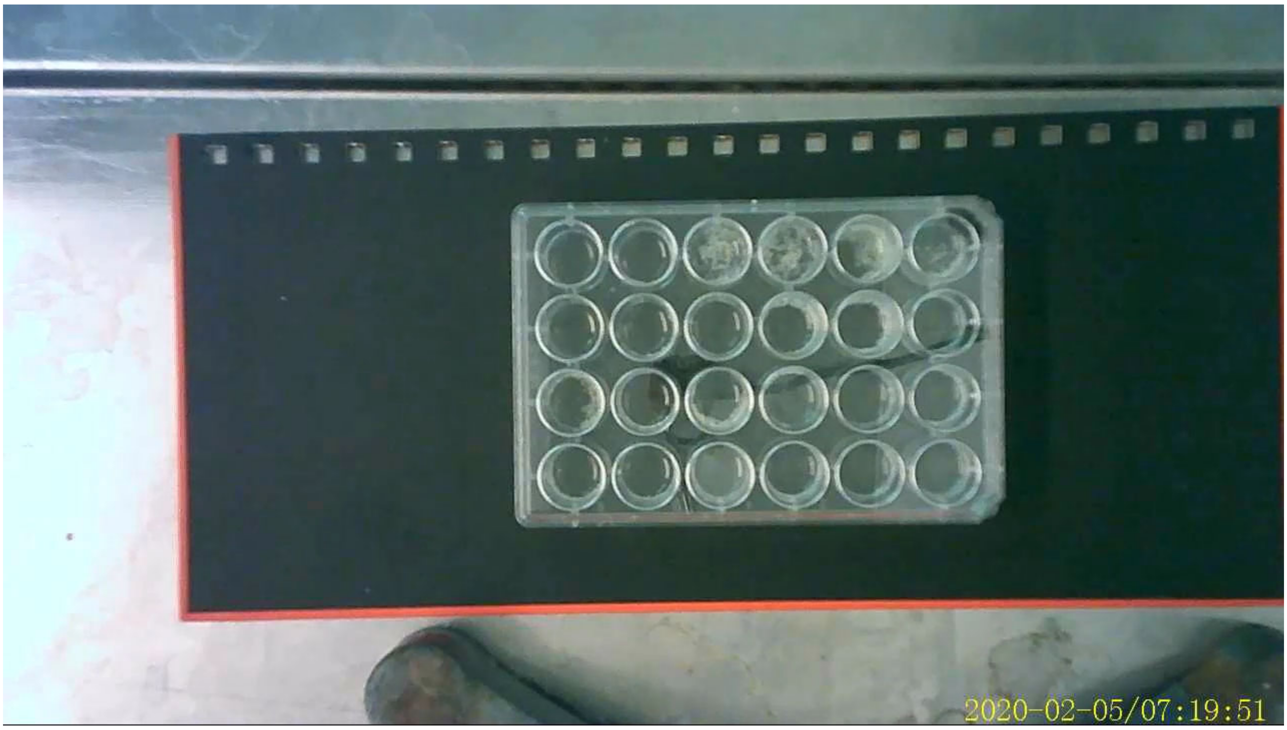

165 min:

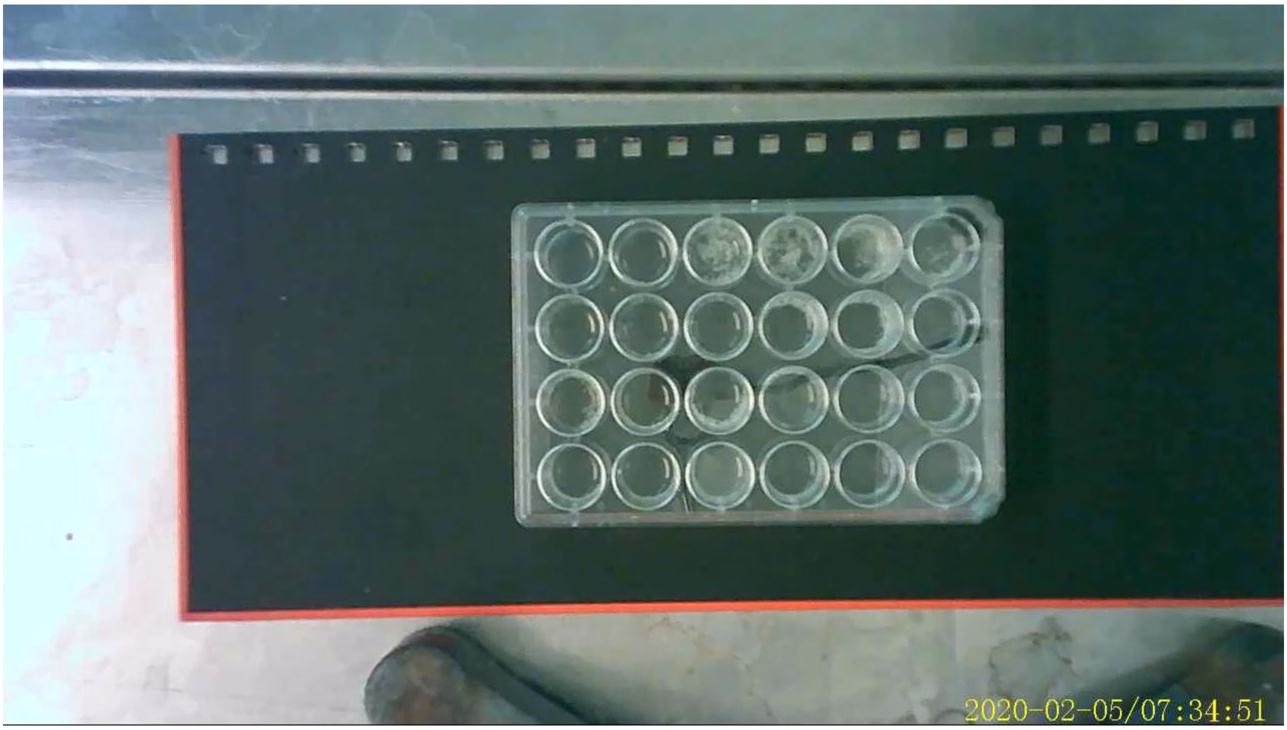

180 min:

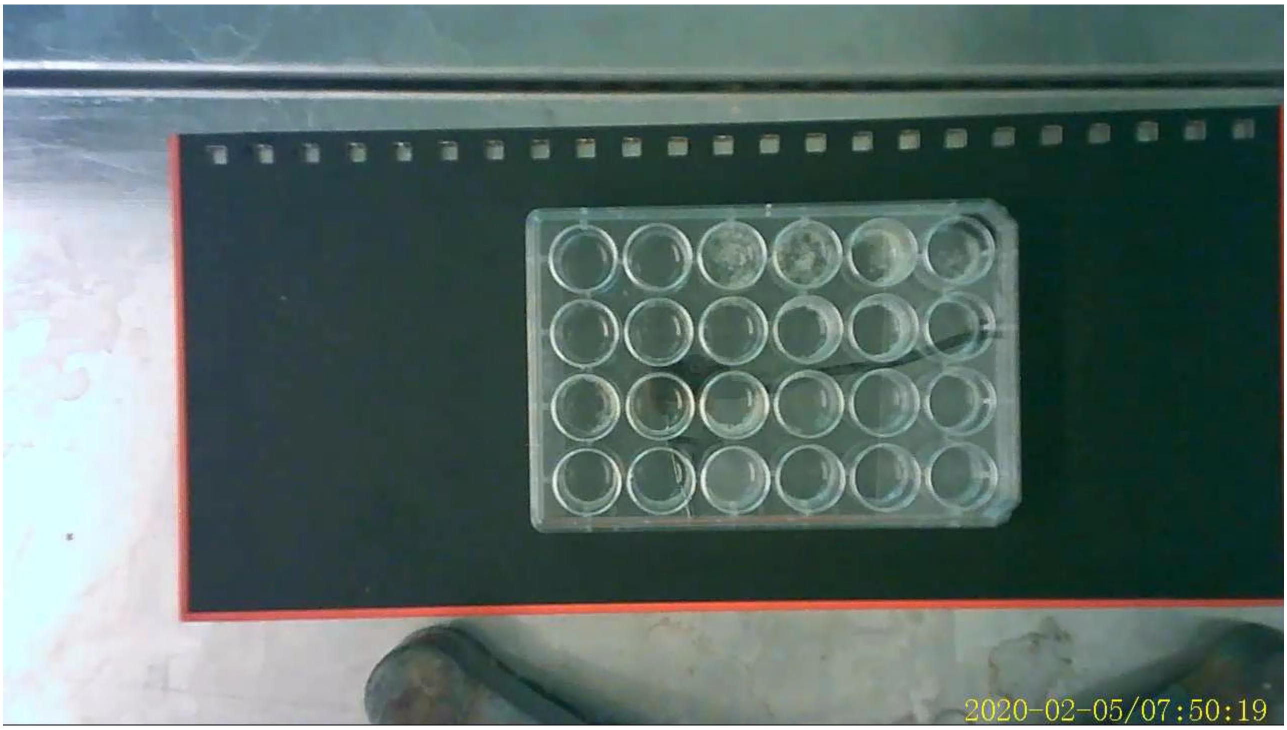

240 min:

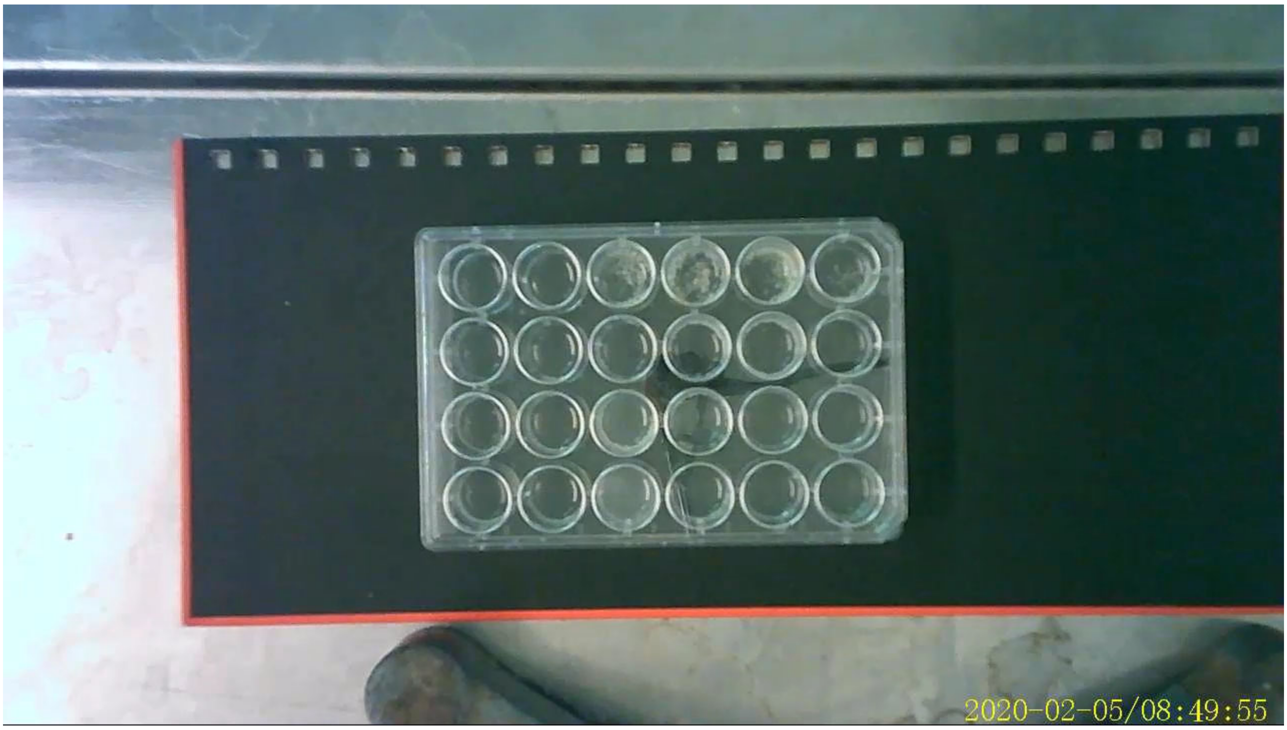

300 min:

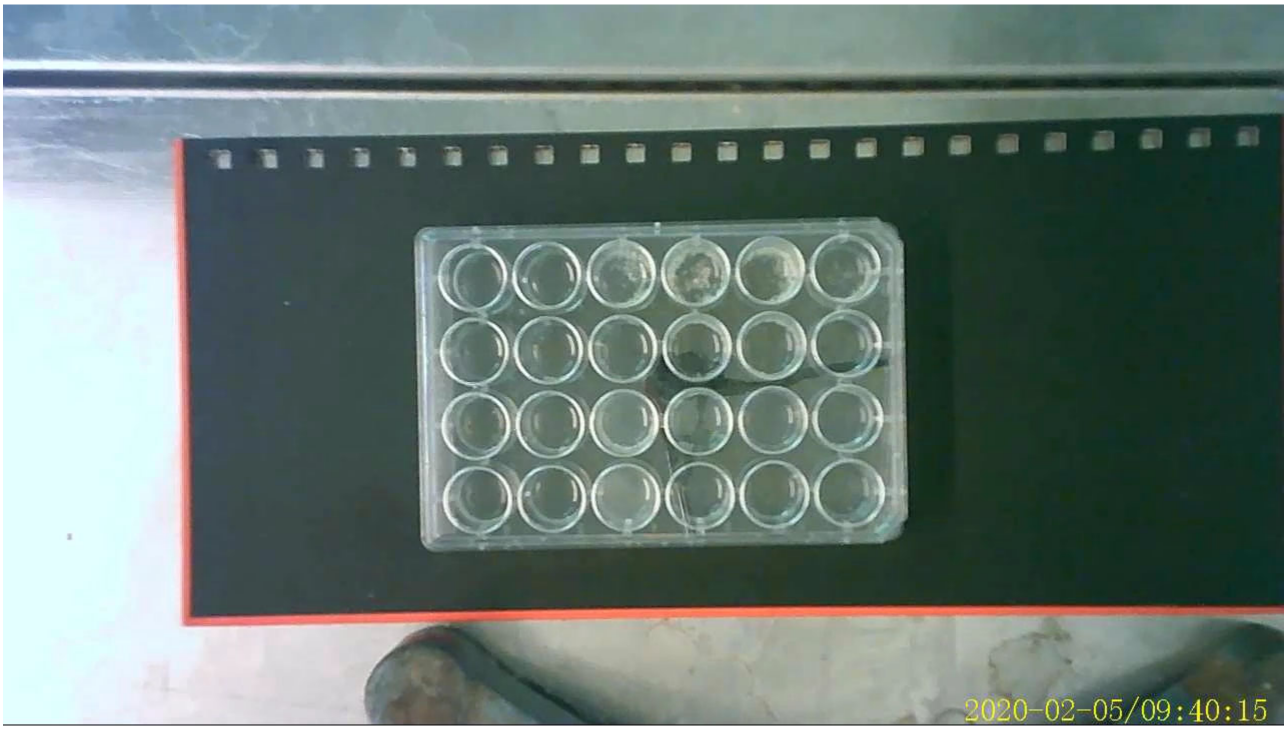

Supplement: Supplementary file 1 [file gels-07-00124-s001.zip › gels-1323594 Supplementary Materials.pdf]
